# LRRC8A Regulates Outer Hair Cell Volume and Electromotility and is Required for Hearing

**DOI:** 10.1002/advs.202410477

**Published:** 2025-07-11

**Authors:** Shengnan Wang, Yuehui Xi, Qiaojun Fang, Sai Shi, Fei Wang, Fuyu Xian, Zhongyang Zhang, Yuxin Yang, Xishuo Jin, Xiaomin Wang, Chen Cao, Hailin Zhang, Nikita Gamper, Zhigang Xu, Haitao Shen, Ping Lv

**Affiliations:** ^1^ Department of Pharmacology The Key Laboratory of Neural and Vascular Biology Ministry of Education The Key Laboratory of New Drug Pharmacology and Toxicology The Hebei Collaboration Innovation Center for Mechanism Diagnosis and Treatment of Neurological and Psychiatric Disease Hebei Medical University Shijiazhuang Hebei 050017 China; ^2^ Shandong Provincial Key Laboratory of Animal Cells and Developmental Biology and Key Laboratory for Experimental Teratology of the Ministry of Education School of Life Sciences Shandong University Qingdao Shandong 266237 China; ^3^ Department of Otolaryngology‐Head and Neck Surgery The Second Affiliated Hospital of Anhui Medical University Hefei 230601 China; ^4^ Department of Medical and Pharmaceutical Informatics Hebei Medical University Shijiazhuang Hebei 050017 China; ^5^ Faculty of Biological Sciences School of Biomedical Sciences University of Leeds Leeds LS2 9JT UK; ^6^ Department of Otolaryngology Head and Neck Surgery the First Hospital of Lanzhou University Lanzhou Gansu 730000 China; ^7^ Lab of Pathology Hebei Medical University Shijiazhuang Hebei 050017 China; ^8^ Hebei Collaborative Innovation Center of Tumor Microecological Metabolism Regulation Affiliated Hospital of Hebei University Baoding Hebei 050017 China

**Keywords:** cell volume regulation, LRRC8, outer hair cells, prestin, VRAC

## Abstract

Damage to the cochlear outer hair cell (OHC) is a major cause of deafness in mammals. OHCs amplify the auditory signals to enhance the sensitivity and sound frequency selectivity of the auditory system. However, detailed mechanisms underlying functional OHC regulation remain unclear. Here, it is demonstrated that volume‐regulated anion channels (VRACs) are essential for OHC function. VRAC subunits are highly expressed in OHCs. Genetic deletion of leucin‐rich repeat containing 8A (LRRC8A), the obligatory VRAC subunit, in hair cells results in hearing loss, whereas re‐expressing LRRC8A in the cochlea restores hearing in LRRC8A‐deficient mice. By employing patch‐clamp electrophysiology, it is shown that VRAC in OHCs is activated by hypotonicity and is involved in regulating cell volume. Furthermore, the nonlinear capacitance (NLC), a measure of electromotility, is decreased in the OHCs from LRRC8A‐deficient mice. Therefore, LRRC8A deficiency compromises electromotility of OHCs and impairs signal amplification. In conclusion, the findings highlight the essential role of VRAC in OHC electromotility and its necessity for auditory function.

## Graphical abstract

This study identifies LRRC8A‐dependent volume‐regulated anion channels (VRACs) as essential for cochlear outer hair cells' electromotility and auditory signal amplification. LRRC8A deficiency disrupts cell volume control, impairs auditory sensitivity, and causes deafness, while targeted LRRC8A re‐expression restores auditory function. These results establish VRAC as a critical molecular regulator of hearing.

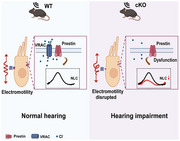

## 1 Introduction

Hearing loss is one of the most prevalent sensory disabilities in humans, and damage to the cochlear outer hair cells (OHCs) induced by noise, aging, or drug toxicity is a major hearing loss cause. Since mature mammalian OHCs do not regenerate, their damage leads to an irreversible decrease in hearing sensitivity.^[^ ^1^ ^]^ Therefore, investigating the molecular mechanisms underlying OHC survival and function is crucial for preventing hearing loss.

Hair cells are the mechanoreceptors responsible for sound perception. There are three rows of OHCs and one row of inner hair cells (IHCs) in the organ of Corti in the mammalian cochlea, each with distinct functions. IHCs receive most of the afferent innervation and transmit auditory information to the central nervous system, while OHCs alter their lengths in response to changes in membrane potential, enhancing the mechanical displacement of the basilar membrane and amplifying auditory signals. This process, known as electromotility, is required for high sensitivity and sharp frequency selectivity in mammals.^[^ ^2^, ^3^, ^4^ ^]^ Mammalian OHC electromotility is mainly mediated by the motor protein Prestin,^[^ ^5^ ^]^ which is abundantly expressed in the lateral membranes of OHCs. Prestin undergoes voltage‐dependent conformational changes that mediate electromotility. However, the continuous cell body stretching during electromotility poses a significant challenge to the shape‐changing capabilities of OHCs, necessitating volume regulation. Thus, identifying volume‐regulated molecules involved in electromotility is essential for understanding OHC amplification.

Cell volume regulation is crucial for cellular homeostasis, metabolism, and signaling.^[^ ^6^ ^]^ Cells respond to osmotic swelling via a regulatory volume decrease (RVD) mechanism by extruding KCl and water from the cytosol.^[^ ^6^, ^7^, ^8^ ^]^ It was reported that the cation channel TMEM63B is involved in Ca^2+^‐dependent RVD in OHCs.^[^ ^9^ ^]^ Ca^2+^ influx through such cation channels may, in turn, activate Ca^2+^‐activated K^+^ channels and/or Ca^2+^‐activated Cl^−^ channels (CaCCs) in response to volume changes.^[^ ^10^, ^11^ ^]^ Osmotic response is also regulated by the volume‐regulated anion channels (VRACs) permeable to Cl^−^ and small organic solutes.^[^ ^12^, ^13^ ^]^ The efflux of Cl^−^ and osmotically active organic molecules (e.g., taurine, glutamate, and myoinositol) through the VRAC drives water outflow, resulting in RVD. Notably, Cl^−^ is also the essential ion supporting OHC electromotility.^[^ ^14^ ^]^ However, the potential role of the VRAC in the functional regulation of OHCs remains unknown.

VRAC is composed of leucin‐rich repeat containing 8 (LRRC8) family proteins (LRRC8A‐E) assembled as hexamers.^[^ ^15^, ^16^, ^17^ ^]^ LRRC8A, the core component of the channel, is the only obligatory subunit of the VRAC. LRRC8A conjugates with at least one of the other LRRC8 family members (LRRC8B‐E) to form a functional channel.^[^ ^16^, ^17^ ^]^ The channel properties vary depending on whether the partner is LRRC8B, C, D, or E.^[^ ^16^, ^17^, ^18^ ^]^ VRAC currents have been recorded in various cell types.^[^ ^19^, ^20^ ^]^ It is activated slowly in response to hypotonic conditions and displays a modest outward rectification. Besides opening by cellular swelling, VRAC is also isovolumetrically activated by intracellular GTPγS, ROS, and Sphingosine‐1‐phosphate (S1P) through various pathways.^[^ ^21^, ^22^, ^23^ ^]^ Consequently, the VRAC is involved in numerous physiological and cellular processes, including hormone secretion, apoptosis, cell proliferation and migration, in addition to cell volume regulation.^[^ ^24^, ^25^ ^]^ For instance, its opening induces depolarization of the β‐cell membrane, which activates voltage‐dependent calcium channels and promotes insulin secretion.^[^ ^26^, ^27^ ^]^ Moreover, glutamate release from astrocytes through VRAC is essential for physiological astrocyte‐neuron communication.^[^ ^19^, ^28^ ^]^ Thus, VRAC is crucial in maintaining multiple cellular functions.

In this study, we demonstrated that VRAC is a major volume‐regulated ion channel in OHCs. Deletion of LRRC8A in hair cells leads to progressive hearing loss in mice. Furthermore, VRAC in OHCs is activated by hypotonicity, and LRRC8A‐deficient OHCs show impaired volume regulation. Importantly, the disruption of LRRC8A resulted in impaired electromotility, indicating that VRAC is an essential contributor to the electromotility of OHCs and is required for auditory function.

## 2 Results

### 2.1 VRAC is Expressed in the Mouse Cochlea

**Figure 1:**
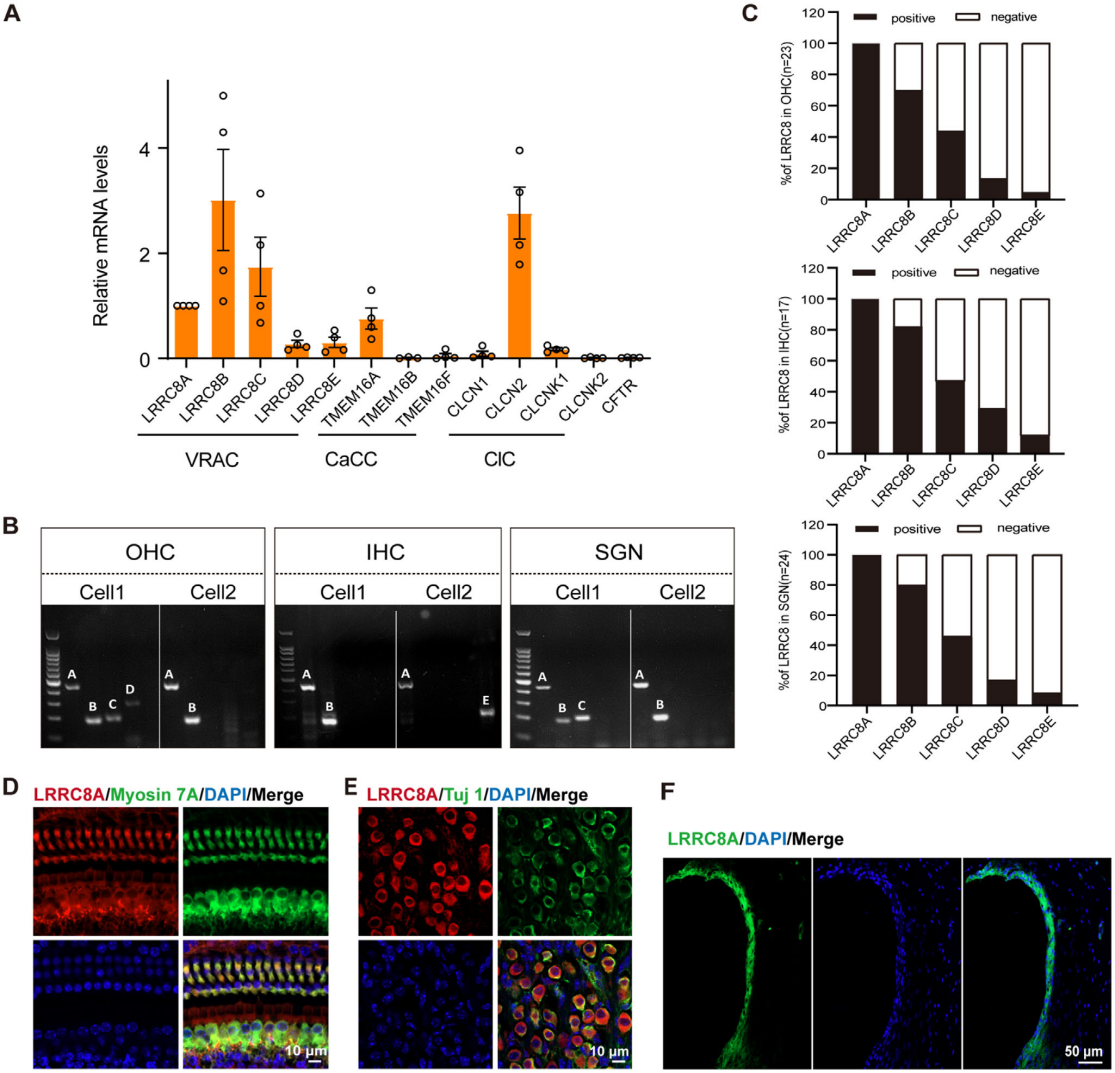
VRAC is expressed in the mouse cochlea. A) Expression of volume‐regulated channel subunits in the 2‐month‐old WT mouse cochlea was examined by real‐time PCR. Data were normalized to LRRC8A and presented as relative expression levels. *n* = 4 pools of total RNA from 8–10 mice. B) Example of single‐cell RT‐PCR analysis of LRRC8 expression in spiral ganglion neurons (SGNs), outer hair cells (OHCs), and inner hair cells (IHCs) of WT mice at P60, P15, and P15, respectively. C) Proportions of cells expressing other LRRC8 subtypes in LRRC8A‐positive OHCs, IHCs, and SGNs. D) LRRC8A (red) co‐localized with Myosin 7A (green) in OHCs and IHCs from 2‐month‐old WT mice. The nuclei were stained with DAPI (blue). E) Expression of LRRC8A (red) in SGNs from 2‐month‐old WT mice. SGNs were stained with anti‐Tuj1, a neuron marker (green). F) Expression of LRRC8A (green) in stria vascularis in the cochlea of 2‐month‐old WT mice.

We first examined the mRNA expression of various volume‐associated anion channels, including the VRAC channel, CaCC channel, voltage‐gated chloride channels (ClC), and cAMP‐activated cystic fibrosis transmembrane conductance regulator (CFTR) anion channel, in the cochlea of 2‐month‐old wild‐type (WT) mice by performing real‐time polymerase chain reaction (PCR). The results showed that TMEM16A and CLCN2 are readily detected in the mouse cochlea (**Figure** **1**A). Intriguingly, all LRRC8 family members are expressed in the cochlea at this age (Figure 1A). Utilizing single‐cell PCR, we examined LRRC8A‐E expression patterns in isolated cochlear hair cells and cultured spiral ganglion neurons (SGNs). The results revealed that LRRC8A is co‐expressed with various VRAC subunits in OHCs, IHCs, and SGNs of WT mice (Figure 1B), suggesting that it may coassemble with other different LRRC8 family members to form the VRAC. Since LRRC8A is an obligatory VRAC subunit, we analyzed the proportion of each subunit in LRRC8A‐positive cells. LRRC8B and LRRC8C were expressed at high rates (Figure 1C), indicating their potential as the major subunits constituting the channels in OHCs, IHCs, and SGNs.

Furthermore, we validated the localization of LRRC8A in the cochlea of WT mice by performing whole‐mount immunostaining using a specific anti‐LRRC8A antibody. Hair cells and SGNs were labelled by immunostaining using antibodies against Myosin 7A (MYO7A) and Tuj1, respectively. The results revealed that LRRC8A is distributed in OHCs and IHCs (Figure 1D). Additionally, SGNs and the stria vascularis also showed LRRC8A expression (Figure 1E,F).

To determine whether LRRC8A expression level changes during development, we examined the relative mRNA levels in the cochlea at different ages ranging from P1 to P60 by performing real‐time PCR. The results revealed that *Lrrc8a* mRNA levels remain stable in the cochlea from P1 to P60 (Figure S1A, Supporting Information). Moreover, immunofluorescence showed that LRRC8A is present in all the cochlear turns at P1, with increased fluorescence intensity in the apical and middle turns at P14, which returned to baseline levels by P21 (Figure S1B–E, Supporting Information).

### 2.2 LRRC8A is Required for Hearing

**Figure 2:**
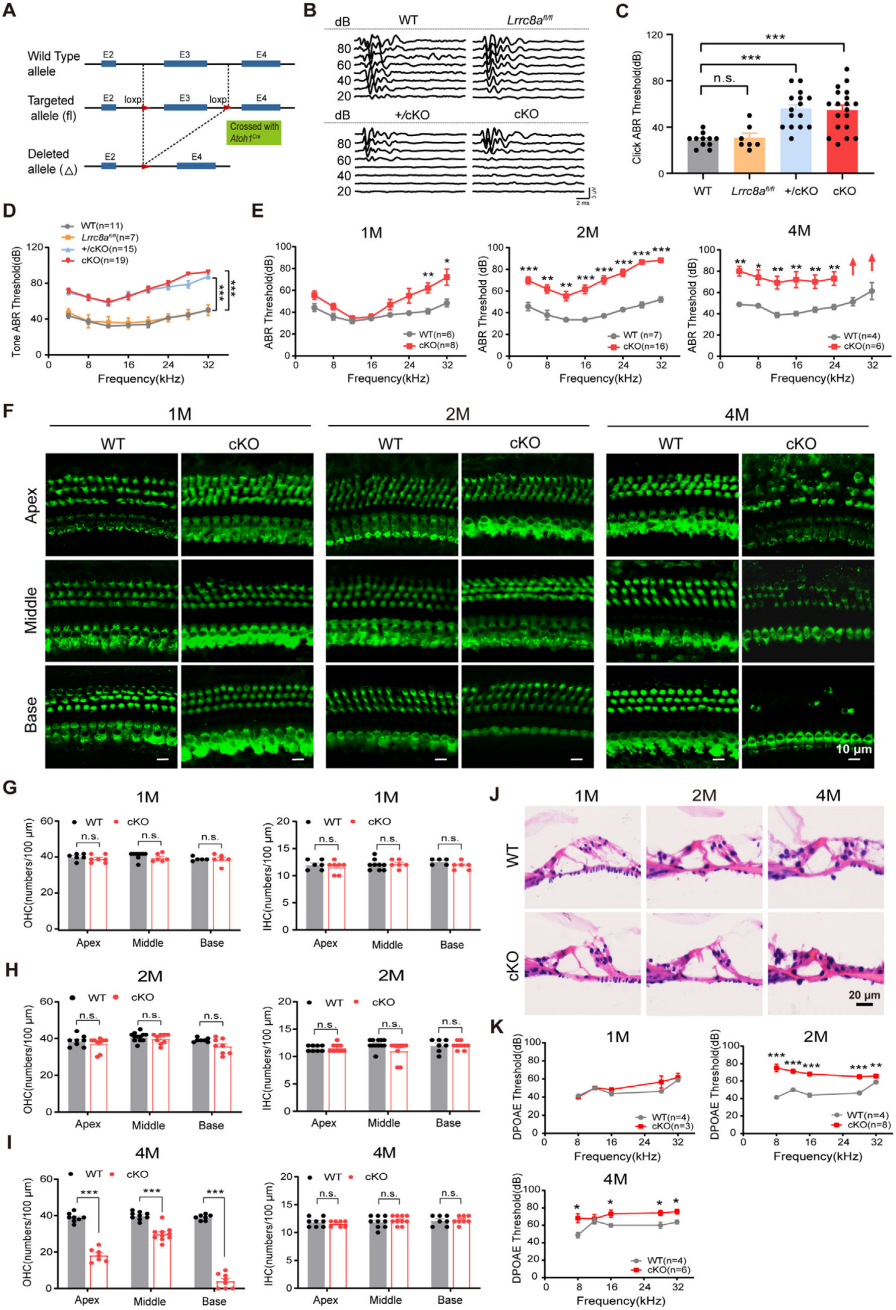
*Lrrc8a* cKO mice exhibit progressive hearing loss and OHC dysfunction. A) Schematic diagram of the strategy for *Atoh1^Cr^* ^e^ *;Lrrc8a^fl/fl^* (cKO) mouse construction. Two LoxP sites were inserted into the alleles of *Lrrc8a*, flanking the coding sequence of exon 3. Cre recombinase expression is driven by the specific *Atoh1* promoter in the cochlea to delete the exon 3 region between the homodromous LoxP sites, resulting in the loss of LRRC8A function in the cochlea. B) Representative ABR waveforms in response to click stimuli in 2‐month‐old WT, *Lrrc8a^fl/fl^*, *Atoh1^Cre^;Lrrc8a^fl/+^* (+/cKO) and *Atoh1^Cre^;Lrrc8a^fl/fl^* (cKO) mice. C,D) ABR thresholds to click (C) or pure tone stimuli (D) were measured in WT, *Lrrc8a^fl/fl^*, +/cKO, and cKO mice at 2 months of age. E) ABR threshold statistics of WT and cKO mice at 1‐, 2‐, and 4‐month‐old in response to pure tone stimuli. F) Myosin 7A‐positive hair cells at the cochlea's apical, middle, and basal regions from 1‐, 2‐, and 4‐month‐old WT and cKO mice. G–I) Quantification of OHCs and IHCs at the cochlea's apical, middle, and basal regions in WT and cKO mice at the age of 1 (G), 2 (H), and 4 months (I). J) The structure of the organ of Corti was examined by HE staining in the basal cochlea of 1, 2, and 4‐month‐old WT and cKO mice. (K) Distortion product otoacoustic emission (DPOAE) threshold measurement of 1‐, 2‐ and 4‐month‐old WT and cKO mice. Data are means ± SEM, ^*^ *p* < 0.05, ^**^ *p* < 0.01, ^***^ *p* < 0.001 by one‐way ANOVA (C) and two‐way ANOVA (D, E, G, H, I, K).

To examine the role of LRRC8A in hearing, we generated cochlea‐specific LRRC8A knockout mice, which disrupt *Lrrc8a* in the hair cells, SGNs, and some supporting cells. Mice with homozygous intronic LoxP sites flanking exon 3 of LRRC8A (*Lrrc8a^fl/fl^*) were crossed with *Atoh1^Cre^* mice (**Figure** **2**A; Figure S2A–D, Supporting Information). The auditory function of 2‐month‐old WT, *Lrrc8a^fl/fl^*, *Atoh1^Cre^;Lrrc8a^fl/+^* (+/cKO), and *Atoh1^Cre^;Lrrc8a^fl/fl^* (cKO) mice was evaluated by measuring the auditory brainstem responses (ABR) to click and pure‐tone stimuli. Both +/cKO and cKO mice exhibited significantly increased ABR thresholds (as compared to WT), indicating hearing impairment (Figure 2B–D). No statistically significant differences were observed in hearing thresholds between WT and *Lrrc8a^fl/fl^* mice (Figure 2B–D) or between WT and *Atoh1^Cre^* mice at the age of 2 months (Figure S2E,F, Supporting Information), indicating that neither the floxed *Lrrc8a* allele nor the *Atoh1*‐driven Cre expression alone affects auditory function.

To examine whether auditory function regulation by LRRC8A is age‐related, we analyzed the ABR thresholds of 1‐, 2‐, and 4‐month‐old cKO mice. Compared with age‐matched WT mice, 1‐month‐old cKO mice displayed significantly elevated ABR thresholds at 28 and 32 kHz, while 2‐month‐old cKO mice exhibited higher ABR thresholds at all frequencies (Figure 2E; Figure S2H,I, Supporting Information). Furthermore, in 4‐month‐old cKO mice, no detectable ABRs were observed at 28 and 32 kHz, suggesting complete deafness at the high‐frequency range (Figure 2E). Taken together, these findings demonstrate that LRRC8A deficiency in the cochlea causes progressive hearing loss in adult mice.

### 2.3 Lrrc8a cKO Mice Displayed Dysfunction of OHCs

To investigate the cellular basis of hearing loss caused by LRRC8A deficiency, we examined the morphology of hair cells, SGNs, and stria vascularis in cKO mice. As assessed via MYO7A immunofluorescence and hematoxylin and eosin (HE) staining, cKO mice exhibited normal OHC morphology in the apical, middle, and basal cochlear turns at 1 and 2 months of age (Figure 2F–J). However, significant OHC degeneration was observed in 4‐month‐old cKO mice (Figure 2F,I,J). In contrast, IHCs, SGNs, and stria vascularis appeared normal in the cochlea up to 4 months of age (Figure 2F,J; Figure S3A–D, Supporting Information). We then performed distortion product optoacoustic emission (DPOAE) measurements to evaluate the function of OHCs in cKO mice. Interestingly, although no OHC degeneration was observed in 2‐month‐old cKO mice, DPOAE thresholds were significantly elevated in cKO mice at this age, indicating OHC dysfunction (Figure 2K). In contrast, *Atoh1^Cre^* mice maintained DPOAE thresholds comparable to WT controls (Figure S2G, Supporting Information). Taken together, these findings suggest that OHC deficiency may be an important contributor to hearing loss in cKO mice.

The *Atoh1^Cre^;Lrrc8a^fl/fl^* mice (cKO) are cochlea‐specific LRRC8A knockout mice, in which *Lrrc8a* is disabled in various cochlear cell types, including hair cells, SGNs, and some supporting cells. To specifically investigate the role of LRRC8A in hair cells, we generated hair‐cell‐specific LRRC8A knockout mice by crossing *Lrrc8a^fl/fl^* with *Gfi1^Cre^* mice (Figure S4A,B, Supporting Information). The *Gfi1^Cre^;Lrrc8a^fl/fl^* mice exhibited specific ablation of LRRC8A in hair cells, with no deletion in SGNs or the stria vascularis (Figure S4C–E, Supporting Information). Consistent with the results obtained with *Atoh1^Cre^;Lrrc8a^fl/fl^* mice, *Gfi1^Cre^;Lrrc8a^fl/+^* and *Gfi1^Cre^;Lrrc8a^fl/fl^* mice similarly exhibited hearing impairment at 2 months of age (Figure S4F–H, Supporting Information). In contrast, *Gfi1^Cre^* mice showed normal hearing thresholds, indistinguishable from WT (Figure S4I,J, Supporting Information). Additionally, the DPOAE thresholds were elevated in 2‐month‐old *Gfi1^Cre^;Lrrc8a^fl/fl^* mice but not in *Gfi1^Cre^* mice, indicating LRRC8A‐dependent OHC dysfunction (Figure S4K,L, Supporting Information). Notably, both *Atoh1^Cre^;Lrrc8a^fl/fl^* and *Gfi1^Cre^;Lrrc8a^fl/fl^* mice exhibited hearing loss at 2 months of age, despite normal hair cell numbers and morphology (Figure 2F,H,J; Figure S4M–O, Supporting Information), suggesting that functional impairment rather than structural defects in OHCs contributes to deafness at this age.

### 2.4 LRRC8A is Required for Swelling‐Activated Currents in OHCs

**Figure 3:**
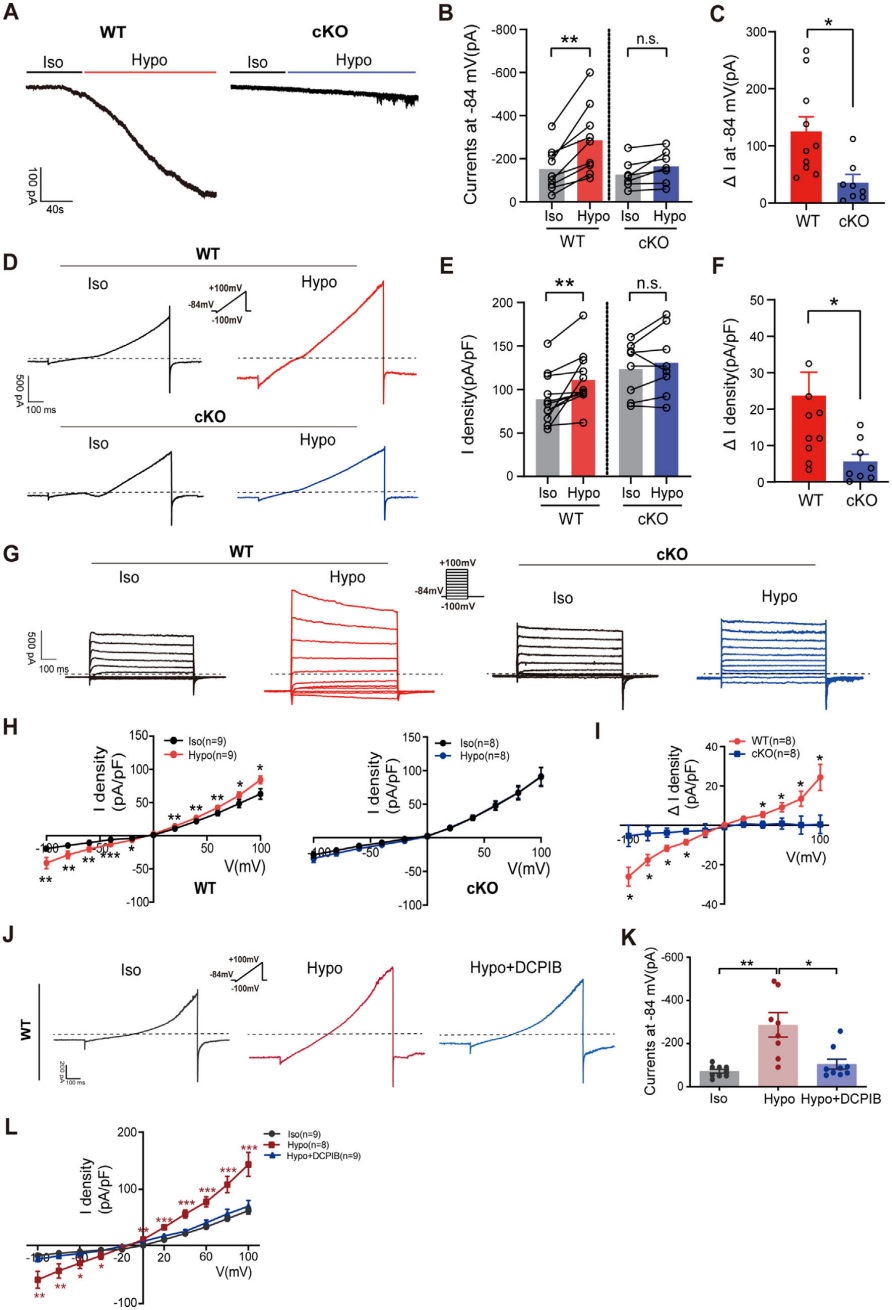
LRRC8A is required for hypotonicity‐induced VRAC currents in OHCs A) Representative current traces induced by Iso (300 mOsm kg^−1^) and Hypo (220 mOsm kg^−1^) solution recorded at ‐84 mV in OHCs from P8‐P12 WT and cKO mice. B) Quantification of current evoked by Hypo solution in WT and cKO OHCs (peak amplitude at ‐84 mV). C) Quantification of changes of Hypo‐induced currents (Δ I) at ‐84 mV in OHCs. D) Representative whole‐cell currents recorded using ramp protocol in Iso and Hypo solutions from OHCs of P8‐P12 WT and cKO mice. E) Quantification of current densities at +100 mV evoked by Hypo solution. F) Quantification of changes of Hypo‐induced currents at +100 mV in OHCs. G) Representative whole‐cell currents recorded by voltage step in OHCs of P8‐P12 WT and cKO mice under Iso and Hypo conditions. H) The current density–voltage relationship in Iso and Hypo conditions. I) The changes of the current density‐voltage relationship in Iso and Hypo conditions. J) Representative whole‐cell currents recorded from OHCs of P8‐P12 WT mice using a ramp protocol under Iso, Hypo, and Hypo+DCPIB (10 µm) conditions. K) Quantification of current at ‐84 mV under Iso, Hypo, and Hypo+DCPIB conditions. L) The current density–voltage relationship in Iso, Hypo, and Hypo+DCPIB conditions. Data are means ± SEM, ^*^ *p* < 0.05, ^**^ *p* < 0.01, ^***^ *p* < 0.001 by Student's *t*‐test (B, C, E, F), one‐way ANOVA (K), and two‐way ANOVA (H, I, L).

Using the whole‐cell voltage‐clamp technique, we recorded currents induced by hypotonicity in OHCs from WT and cKO mice at P8–P12. A pronounced swelling‐activated inward current was elicited at ‐84 mV holding potential in WT OHCs by perfusion with a hypotonic solution (220 mOsm kg^−1^) compared with an isotonic solution (300 mOsm kg^−1^) (**Figure** **3**A–C). In contrast, hypotonicity‐induced currents were significantly reduced in OHCs of cKO mice (Figure 3A–C). We further recorded the currents under isotonic (300 mOsm kg^−1^) and hypotonic (220 mOsm kg^−1^) conditions in OHCs from WT and cKO mice using ramp (Figure 3D–F) and voltage‐step protocols (Figure 3G–I). Similarly, perfusion of a hypotonic solution quickly elicited swelling‐activated currents in OHCs of WT mice, whereas the response of cKO OHCs to hypotonic stimulus was nearly completely abolished (Figure 3D–I), indicating that LRRC8A is required for the swelling‐activated currents in OHCs.

Furthermore, application of 4‐(2‐butyl‐6, 7‐dichloro‐2‐cyclopentyl‐indan‐1‐on‐5‐yl) oxobutyric acid (DCPIB), a specific VRAC inhibitor, significantly attenuated the swelling‐activated currents in WT OHCs (Figure 3J–L). Consistently, in the cultured House Ear Institute‐Organ of Corti 1 (HEI‐OC1) cells, we observed similar pharmacological inhibition, with DCPIB treatment effectively suppressing hypotonicity‐induced currents (Figure S5A–G, Supporting Information). These data collectively demonstrate that VRAC serves as the primary contributor to the swelling‐activated currents in OHCs.

### 2.5 LRRC8A is Crucial for OHC Volume Regulation

**Figure 4:**
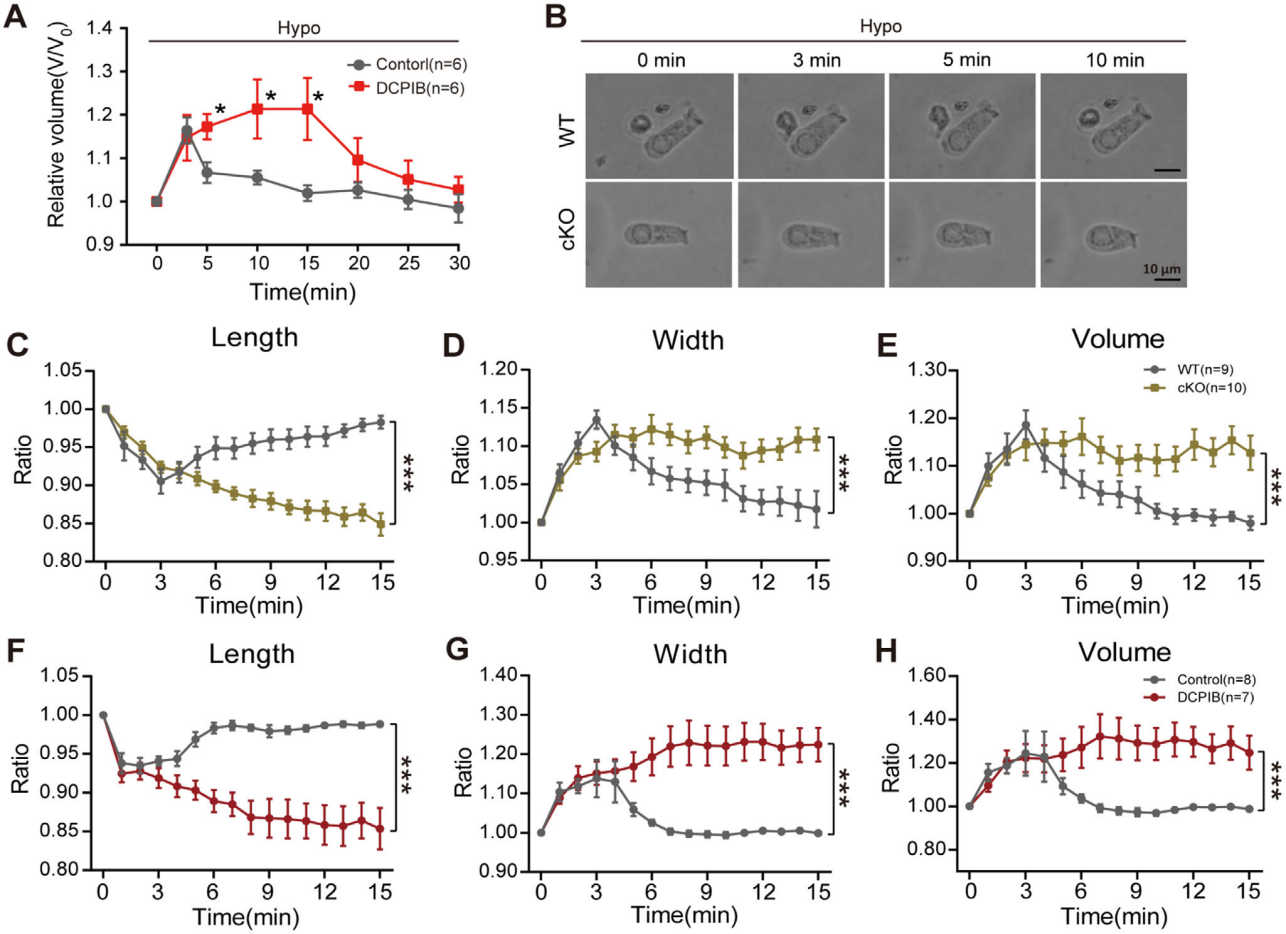
LRRC8A is required for volume regulation of OHCs. A) DCPIB (10 µm) inhibits the regulatory volume decrease in HEI‐OC1 cells. B) Images of OHCs from P10‐P14 WT and cKO mice under isotonic conditions (300 mOsm kg^−1^), followed by treatment with hypotonic (285 mOsm kg^−1^) solution at 0, 3, 5, and 10 min. C–E) The changes in length (C), width (D), and volume (E) of isolated OHCs from P10‐P14 WT and cKO mice treated with hypotonic solution. F–H) The changes in length (F), width (G), and volume (H) of isolated OHCs from P10‐P14 WT mice treated with a hypotonic solution in the presence of DCPIB (10 µm). Data are means ± SEM, ^*^ *p* < 0.05, ^***^ *p* < 0.001 by two‐way ANOVA.

To clarify the mechanistic role of VRAC in cellular volume homeostasis, we assessed its regulatory function under both isotonic and hypotonic conditions. We first examined hypotonic stress‐induced volume regulation in HEI‐OC1 cells. Under hypotonic stress, control HEI‐OC1 cells rapidly swelled, initiating RVD, and returned to normal volume (**Figure** **4**A). In contrast, DCPIB‐treated cells demonstrated significantly larger volumes than controls, indicating that VRAC inhibition blocked RVD (Figure 4A). We further assessed volume regulation in acutely dissociated OHCs under hypotonic stress. The results showed that WT OHCs presented much milder shape changes compared to cKO OHCs (Figure 4B). Noticeably, the cKO OHCs exhibited significant length reduction and width increase compared with WT OHCs (Figure 4C,D), indicating impaired volume regulation due to LRRC8A deletion (Figure 4E).

We further investigated the volume regulation in OHCs of *Gfi1^Cre^;Lrrc8a^fl/+^* mice. Notably, these mice also exhibited impaired RVD (Figure S6A–E, Supporting Information), demonstrating that partial LRRC8A expression in OHCs is insufficient to maintain normal volume homeostasis. Similarly, DCPIB‐treated WT OHCs displayed increased width and volume as well as decreased length when compared to controls (Figure 4F–H), confirming LRRC8A's critical role in OHC volume regulation.

### 2.6 LRRC8A is Essential for the Electromotility Function of OHCs

**Figure 5:**
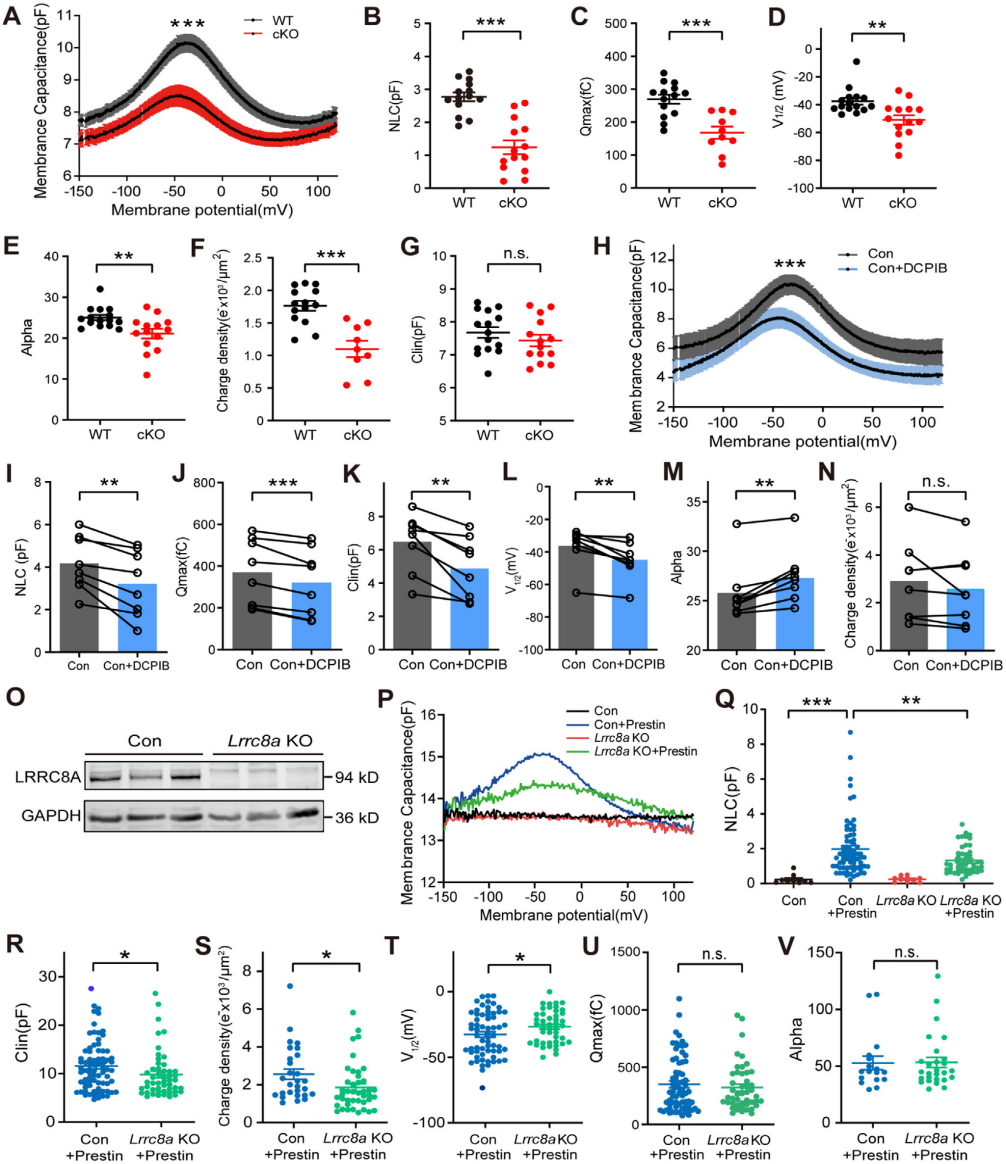
LRRC8A is essential for the electromotility function of OHCs. A) Average capacitance as a function of voltage in OHCs from WT and cKO mice at P8–P12. B) Quantification of NLC in OHCs from WT and cKO mice. C–G) Electromotility parameters in OHCs from WT and cKO mice, total charge moved (Q_max_, C), voltage at peak capacitance (V_1/2_, D), slope factor of voltage dependence (α, E), charge density (F), and linear capacitance (C_lin_, G). H) Average capacitance as a function of voltage was measured in WT OHCs from control and DCPIB‐treated (10 µm) groups. I) Quantification of NLC in control versus DCPIB‐treated OHCs. (J–N) Electromotility parameters in control and DCPIB‐treated OHCs, Q_max_ J), C_lin_ K), V_1/2_ L), α M), and charge density N). O) Western blot using an anti‐LRRC8A antibody was performed to examine LRRC8A expression in control and *Lrrc8a* KO HEK293A cells. GAPDH was included as an internal control. P) Voltage‐dependent capacitance of HEK293A cells from four groups, WT (Con), WT overexpressing Prestin (Con+Prestin), *Lrrc8a* KO, and *Lrrc8a* KO overexpressing Prestin (*Lrrc8a* KO+Prestin). Q) Quantification of NLC in HEK293A cells from different groups. R–V) Electromotility parameters in Con+Prestin and *Lrrc8a* KO+Prestin cells, C_lin_ (R), charge density (S), V_1/2_ (T), Q_max_ (U), and α (V). Data are means ± SEM, ^*^ *p* < 0.05, ^**^ *p* < 0.01, ^***^ *p* < 0.001 by Student's *t*‐test (B‐G, I‐N, R‐V), one‐way ANOVA (Q), and two‐way ANOVA (A, H).

We next investigated the role of LRRC8A in regulating OHC electromotility. OHCs isolated from P8 to P12 mice were analyzed using whole‐cell patch clamp. Nonlinear capacitance (NLC) was measured to assess the electromotility function of OHCs.^[^ ^29^, ^30^ ^]^ The results revealed that NLC was significantly lower in the OHCs from cKO mice compared to WT (**Figure** **5**A,B). In addition, NLC parameters, including the total charge moved (Q_max_), voltage at peak capacitance (V_1/2_), charge density, and the slope factor of voltage dependence (α), were also decreased in cKO OHCs (Figure 5C–F), suggesting a decline of electromotility in cKO mice. In contrast, linear capacitance (C_lin_) was not significantly different between WT and cKO mice (Figure 5G). We further measured NLC in OHCs from *Atoh1^Cre^;Lrrc8a^fl/+^* and *Gfi1^Cre^;Lrrc8a^fl/+^* mice. Compared to WT, OHCs from both genotypes exhibited significantly reduced NLC (Figure S7A–N, Supporting Information), indicating impaired electromotility, which may contribute to their observed hearing deficits. Moreover, to confirm the effect of VRAC on electromotility, we inhibited VRAC in WT OHCs using DCPIB. DCPIB‐treated OHCs from WT mice exhibited significant reductions in NLC, Q_max_, C_lin_, and V_1/2_ (Figure 5H–L). Interestingly, the α was elevated in DCPIB‐treated cells (Figure 5M), and the charge density was not significantly different between control and DCPIB‐treated WT OHCs (Figure 5N). We further examined the effect of DCPIB on the electromotility of OHCs in cKO mice. DCPIB treatment did not significantly alter the NLC or other parameters related to electromotility in cKO OHCs (Figure S8A–G, Supporting Information), indicating that DCPIB has no direct effect on Prestin, the molecular motor essential for OHC electromotility.

For further validation, we overexpressed Prestin in the Human embryonic kidney (HEK293A) cells that express endogenous VRAC (Figure 5O). The control cells (Con) did not exhibit NLC, whereas cells transfected with Prestin (Con+Prestin) displayed significant NLC (Figure 5P,Q). Subsequently, we transfected Prestin into *Lrrc8a* KO HEK293A cells, in which *Lrrc8a* was knocked out using the CRISPR/Cas9 technique (Figure 5O). The NLC was significantly decreased in *Lrrc8a* KO cells transfected with Prestin (*Lrrc8a* KO+Prestin) compared to Con+Prestin cells (Figure 5P,Q). The C_lin_ and charge density were also reduced in *Lrrc8a* KO+Prestin cells, although the V_1/2_ was elevated, while Q_max_ and α remained unchanged (Figure 5R–V). This confirms that LRRC8A is essential for electromotility function.

Electromotility mediated by conformation changes of Prestin depends on the membrane potential of the OHCs. VRAC activity may affect the membrane potential.^[^ ^31^ ^]^ Therefore, we recorded the resting membrane potential of OHCs from WT and cKO mice at P7 and P13. The resting membrane potential was unchanged between WT and cKO mice (Figure S9A,B, Supporting Information). Additionally, outward currents showed no significant differences between these mice (Figure S9C–E, Supporting Information). These data indicated that LRRC8A deficiency does not affect the resting membrane potential of OHCs under basal conditions.

### 2.7 Loss of LRRC8A Affects Prestin Expression in OHCs

To elucidate the mechanisms by which LRRC8A regulates electromotility, we examined its spatial relationship with Prestin. Immunofluorescence using specific anti‐LRRC8A antibody demonstrated that LRRC8A was widely expressed in the cytoplasm and plasma membrane of OHCs and therefore partially overlapped with Prestin (Figure S10A,B, Supporting Information). We further analyzed Prestin expression in cKO mice. At 2 months of age, *Prestin* mRNA levels were significantly higher in cKO cochlear tissues compared to WT, suggesting a potential compensatory response to LRRC8A deletion (Figure S10C, Supporting Information). Additionally, disorganization of Prestin was observed alongside the OHC degeneration in cKO mice at an older age (Figure S10D, Supporting Information).

### 2.8 Re‐Expression of LRRC8A in OHCs Rescues Hearing of Lrrc8a cKO Mice

**Figure 6:**
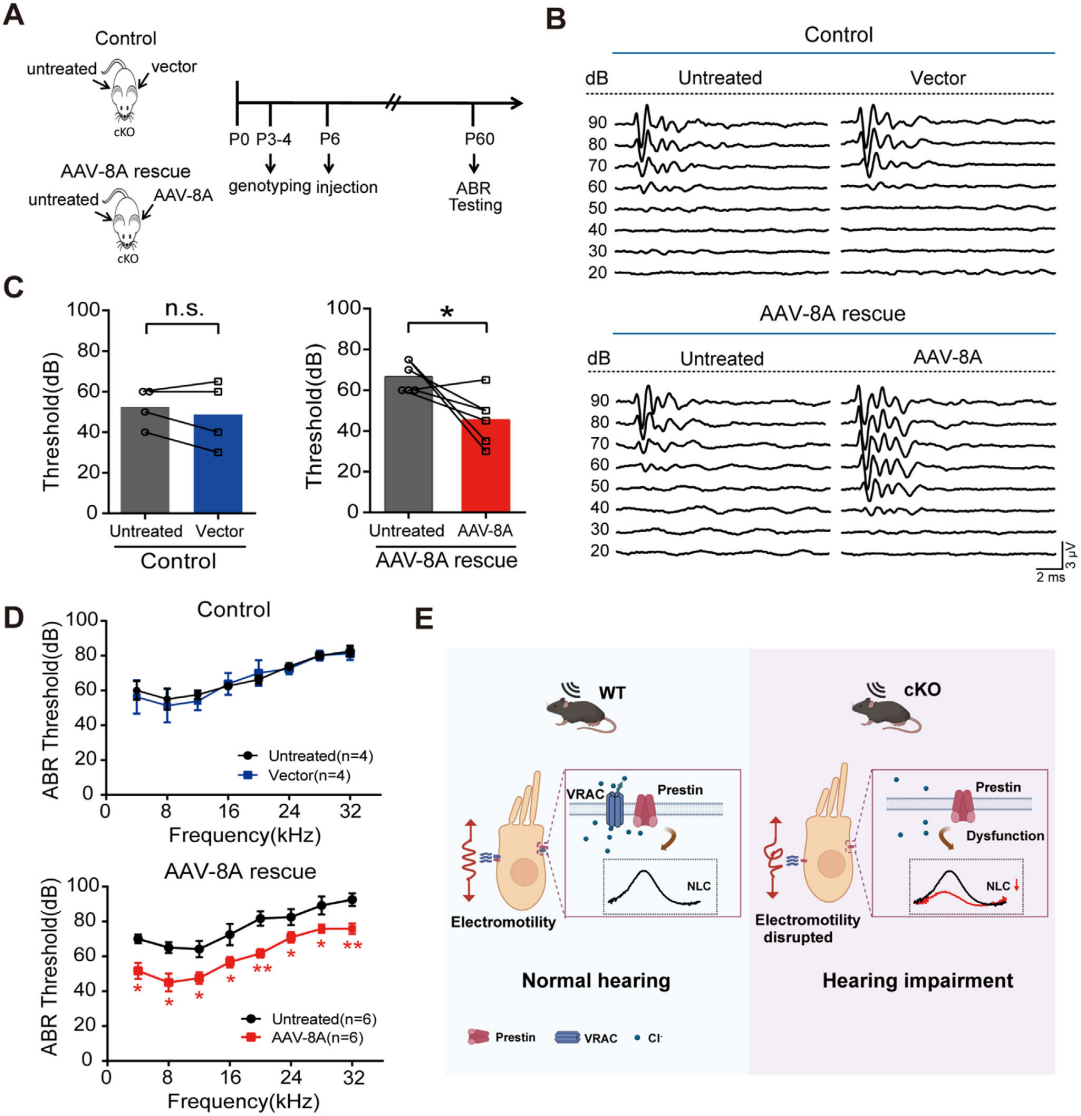
Re‐expression of LRRC8A in OHCs rescues hearing of cKO mice. A) Timeline for rescue experiment in cKO (*Atoh1^Cre^;Lrrc8a^fl/fl^*) mice. Mice in the control group were injected with AAV7m8‐CAG‐P2a‐GFP (vector) in the left ear, and the right ear was left untreated. Mice in the AAV‐8A rescue group were injected with AAV7m8‐CAG‐Lrrc8a‐P2a‐GFP (AAV‐8A) in the left ear, and the right ear was left untreated. B) Representative ABR waveforms in response to click stimuli in the mice in control and AAV‐8A rescue groups. C,D) ABR thresholds to click (C) and pure tone stimuli (D) in control and AAV‐8A rescue mouse groups. E) Schematic depicting the proposed role of VRAC in hearing. Data are means ± SEM, ^*^ *p* < 0.05, ^**^ *p* < 0.01 by Student's *t*‐test (C) and two‐way ANOVA (D).

To investigate whether reintroducing LRRC8A back into OHCs could restore hearing in *Lrrc8a* cKO (*Atoh1^Cre^;Lrrc8a^fl/fl^*) mice, we delivered AAV‐LRRC8A virions into the cochlea of *Lrrc8a* cKO mice at P6 and performed ABR measurement at P60 (**Figure** **6**A). In control experiments, the cKO mice were injected with AAV7m8‐CAG‐P2a‐GFP (vector) in the left ear, while the right ear was untreated. For the AAV‐8A rescue group, cKO mice were injected with AAV7m8‐CAG‐Lrrc8a‐P2a‐GFP (AAV‐8A) in the left ear, with the right ear untreated (Figure 6A). Compared to the untreated ear, the ABR thresholds of the AAV‐8A‐rescue injected ear were significantly decreased, indicating an improvement in hearing (Figure 6B–D). The thresholds in the vector‐injected ear of control mice did not differ from those in the untreated ear (Figure 6B–D).

## 3 Discussion

Previous studies demonstrated that VRAC participates in diverse cellular processes, such as volume regulation, osmolyte transport, and neurotransmitter release across various cell types.^[^ ^24^, ^25^ ^]^ However, despite expression in OHCs, the physiological function of VRAC in the auditory system remains unclear. This study provides several lines of evidence uncovering the functional significance of VRAC in OHC physiology and hearing. We found that VRAC is essential for regulatory volume decrease after hypotonic swelling and critically regulates OHC electromotility, highlighting its significant role in hearing (Figure 6E).

### 3.1 VRAC Subunits are Expressed in Multiple Cell Types in the Cochlea

The regulation of cell volume is critical for cell function and survival. In mammals, anion channels associated with changes in cell volume can be divided into two categories, volume‐activated and volume‐associated anion channels.^[^ ^32^ ^]^ Volume‐activated anion channels are directly activated by cell swelling, including VRAC, maximal anion channels (MAC or Maxi‐Cl), and ClC.^[^ ^32^ ^]^ Volume‐associated anion channels, which are not directly activated by changes in cell volume, are facultatively implicated in changes in cell volume. These include cAMP‐activated CFTR anion channels, CaCC, and acid‐sensitive outwardly rectifying anion channels.^[^ ^32^ ^]^ We observed that all the LRRC8 family members (LRRC8A‐E), TMEM16A/CaCC, and CLCN2/ClC are expressed in the cochlea, suggesting that volume regulation may play a role in auditory physiology.

To form a functional VRAC, LRRC8A must heteromultimerize with the other LRRC8 subunits, giving rise to channels with different properties. For instance, LRRC8A/C or LRRC8A/D heteromers are inhibited by chloramine‐T oxidation, whereas LRRC8A/E heteromers are activated by chloramine‐T oxidation.^[^ ^33^ ^]^ We examined the expression patterns of VRAC in OHCs, IHCs, and SGNs using single‐cell PCR and found that the expression of LRRC8A, B, and C was significantly higher than that of LRRC8D and LRRC8E in these cells, suggesting that LRRC8A, B, and C are the major subunits constituting VRAC channels in the cochlea. Even within the same cell type, LRRC8 could be assembled in different ways, indicating combinatorial diversity in VRAC expression in cochlear cells, which may result in functional variation. This finding is consistent with a recent study by Knecht et al.^[^ ^34^ ^]^

### 3.2 Loss of LRRC8A in Hair Cells Causes Hearing Loss

LRRC8A is an obligatory subunit of VRAC, and its deletion eliminates the VRAC function.^[^ ^16^ ^]^ We used mice in which *Lrrc8a* was specifically knocked out in the cochlea to study the role of VRAC in hearing. We first generated *Atoh1^Cre^;Lrrc8a^fl/fl^* mice (cKO) by crossing *Atoh1^Cre^* mice with *Lrrc8a^fl/fl^* mice. *Atoh1^Cre^* is expressed in hair cells, SGNs, and some supporting cells of the cochlea.^[^ ^35^ ^]^ When *Lrrc8a* was specifically knocked out in these cells, the mice exhibited progressive hearing loss. To further clarify the role of VRAC in hair cells, we also generated hair cell‐specific *Lrrc8a* knockout mice by crossing *Lrrc8a^fl/fl^* mice with *Gfi1^Cre^* mice expressing Cre in IHCs and OHCs.^[^ ^36^ ^]^ Consistent with the findings in *Atoh1^Cre^;Lrrc8a^fl/fl^* mice, *Gfi1^Cre^;Lrrc8a^fl/fl^* mice displayed hearing impairment, suggesting that the VRAC in hair cells is a significant contributor to hearing. However, the effects of VRAC on other cell types during hearing cannot be dismissed. For example, Knecht et al. demonstrated LRRC8A‐E expression in multiple inner ear cell types using knock‐in mice.^[^ ^34^ ^]^ Their study revealed that disruption of LRRC8A in the inner ear caused deafness with concomitant loss of Kir4.1 channels in stria vascularis and reduced endocochlear potential, suggesting VRAC's regulatory role in stria vascularis.^[^ ^34^ ^]^ Additionally, *Sox10^Cre^;Lrrc8a^fl/fl^ *mice in Knecht's study exhibited earlier‐onset hearing loss (3 weeks).^[^ ^34^ ^]^ This phenotypic divergence may stem from multicell LRRC8A ablation in their mice, where synergistic effects accelerate hearing loss progression. These complementary findings underscore the pivotal role of LRRC8A in auditory processing and highlight multiple mechanisms involved.

Notably, our data demonstrated that the heterozygotes of these two *Lrrc8a* knockout mouse lines also showed impaired OHC electromotility and hearing loss. We hypothesize that VRAC may be crucial for maintaining OHC's function and that other channels may be unable to fully compensate for a reduction in VRAC. Consequently, heterozygous mice continue to manifest a deafness phenotype despite retaining some VRAC.

OHC degeneration onset in our knockout mice was not observed until 4 months of age; however, hearing impairment was evident at 2 months of age. These findings indicate that the underlying cause of hearing loss in mice may be OHC dysfunction rather than degeneration. Our DPOAE data further support the finding that cKO mice display impaired OHC function at 2 months of age. As the dysfunction progresses, OHC undergoes degeneration, resulting in progressive hearing loss in mice.

### 3.3 VRAC is a Major Swelling‐Activated Ion Channel in OHCs

Studies have demonstrated that the turgor pressure of OHCs exerts a profound influence on the gain of the cochlear amplifier in both in vitro and in vivo systems.^[^ ^37^, ^38^ ^]^ However, the molecular mechanisms underlying this regulatory process have remained elusive. We detected expression of several swelling‐activated ion channels in the cochlea, including VRAC, CLC‐2, and CaCC, but our electrophysiological results revealed that VRAC serves as the predominant channel responsible for OHC volume regulation in hypotonic conditions. Genetic deletion of LRRC8A significantly reduced swelling‐activated currents in OHCs. Furthermore, OHCs lacking LRRC8A or treated with DCPIB failed to maintain cell shape under hypotonic stimuli, providing unequivocal evidence for the critical role of LRRC8A in OHC volume regulation. Collectively, our findings establish the molecular basis of OHC volume regulation and its role in turgor pressure‐mediated cochlear amplification.

Notably, VRAC activation is slow under hypotonic conditions. However, the high‐frequency OHC motility may require fast recovery of cell shape. Thus, we speculate that the involvement of VRAC in OHC electromotility is not directly related to its volume regulation function. It was reported that acoustic stimulations in the absence of TMEM63B result in a cumulative osmotic imbalance leading to swelling‐dependent cell death.^[^ ^9^ ^]^ Consistently, our data show that LRRC8A deletion leads to impaired OHC volume regulation and OHC degeneration, suggesting that chronic dysfunction of volume regulation may contribute to OHC degeneration.

VRAC is a nonselective anion channel. It not only allows the passage of Cl^−^, I^−^, HCO_3_^−^, and Br^−^, but is also permeable to other organic osmolytes such as taurine, glutamate, and gluconate.^[^ ^19^, ^39^ ^]^ Recently, VRAC has been proposed to be permeable to ATP in spinal microglia, which contributes to neuropathic pain.^[^ ^40^ ^]^ The composition of LRRC8 subunits determines the permeability profile of the channel.^[^ ^41^ ^]^ Although our study characterized the properties of VRAC‐mediated Cl^−^ currents, it did not determine the permeability of other anions in the OHCs as well as their possible physiological consequences, which await further investigation.

### 3.4 VRAC is Crucial for the Electromotility of OHCs

The electromotility of OHCs is the basis for signal amplification during hearing in mammals. Prestin is encoded by the *SLC26A5* gene and is responsible for OHC electromotility. Genetic ablation of *Prestin* in mice leads to a loss of OHC somatic electromotility, accompanied by a significant reduction in hearing sensitivity ranging from 40 to 60 dB.^[^ ^42^ ^]^ In contrast, our cKO mice exhibited a less severe hearing impairment, suggesting that the absence of LRRC8A compromises Prestin function rather than abolishes it entirely.

Notably, Yamashita et al. reported that partial reduction of OHC electromotility can preserve normal hearing sensitivity within the 4–22 kHz frequency range.^[^ ^43^ ^]^ This is consistent with our observations in 1‐month‐old cKO mice, which exhibited normal hearing at these frequencies, suggesting that the initial decline in electromotility during early development may not suffice to induce severe hearing impairment. Furthermore, we speculate that LRRC8A knockout may trigger compensatory mechanisms during early developmental stages, delaying hearing loss. However, with aging progression, the persistent absence of LRRC8A likely leads to progressive failure of these compensatory mechanisms, resulting in elevated ABR thresholds across all tested frequencies. Additionally, Prestin dysfunction itself also contributes to progressive OHC degeneration and hearing impairment,^[^ ^44^ ^]^ a phenotype consistent with our findings in 4‐month‐old cKO mice.

For our electromotility assessments, we used P8‐P12 mice rather than adults due to technical limitations. The progressive cochlear ossification in adult mice creates challenges for basilar membrane isolation, whereas younger mice allow for easier tissue preparation and better OHC activity preservation. Importantly, our data demonstrate comparable LRRC8A expression levels between neonatal and adult OHCs, and Prestin is known to be functionally expressed from P5 to P6,^[^ ^29^, ^45^, ^46^ ^]^ supporting the relevance of our findings in P8‐P12 mice for understanding the regulatory role of LRRC8A in Prestin function.

Recent studies emphasize that Prestin motor kinetics are more critical for cochlear function than changes in its expression.^[^ ^47^, ^48^ ^]^ The Cl^−^ is the key ion to support Prestin's fast motor kinetics.^[^ ^14^ ^]^ It triggers a voltage‐dependent conformational change in Prestin by binding to the transmembrane region.^[^ ^49^, ^50^, ^51^ ^]^ The structure of Prestin, including its Cl^−^ binding sites, was identified using cryoelectron microscopy,^[^ ^51^, ^52^, ^53^ ^]^ further confirming that Cl^−^ is indispensable for normal Prestin function. However, the molecular mechanism of Prestin modulation by Cl^−^ has not been fully understood. Prestin belongs to the SLC26 anion transporter family. However, its anion transport capacity is very weak and of minimal physiological importance for OHC function and maintenance.^[^ ^48^ ^]^ Previous studies revealed minimal contributions of the ClC and CFTR channels to the Cl^−^ conductance of OHCs.^[^ ^54^ ^]^ Instead, Rybalchenko and Santos‐Sacchi reported the existence of a nonselective, stretch‐sensitive channel within the lateral plasma membrane that can be inhibited by volume‐regulated channel blockers.^[^ ^54^ ^]^ They also reported that intracellular Cl^−^ oscillations near Prestin may drive motor protein transitions.^[^ ^54^ ^]^ However, the molecular identity of this channel was not determined at the time. Our present data showed that LRRC8A is expressed on the OHC basolateral membrane and is required for electromotility of OHCs. We propose that VRAC could be the channel involved in regulating Prestin by allowing the passage of Cl^−^. Further research is guaranteed to clarify the detailed underlying mechanisms.

In conclusion, we discovered a dual role of VRAC in OHCs. First, it regulates OHC volume. Second, VRAC is involved in OHC electromotility. Therefore, our findings reveal a vital role of the VRAC in the OHC‐mediated amplification of auditory signals and provide new evidence for elucidating the mechanism of the OHC processing of auditory signals.

## 4 Experimental Section

### Animals

All experimental animal protocols (2 024 219) were performed per the Animal Care and Ethics Committee of Hebei Medical University, China. C57BL/6 mice were purchased from Beijing Vital River Laboratory Animal Technology Co., Ltd. *Lrrc8a^flox/flox^* mice were from Cyagen (NO. S‐CKO‐17808). *Atoh1^Cre^* and *Gfi1^Cre^* mice were maintained and genotyped as previously reported.^[^ ^35^, ^36^ ^]^ All mice were bred in‐house under a 12:12 light‐dark cycle. Mouse‐tail genotyping was performed using the primers listed in Table S1 (Supporting Information).

### Auditory Brainstem Responses (ABR) and Distortion Product Otoacoustic Emissions Measurements (DPOAE)

The ABR and DPOAE measurements were performed as previously described.^[^ ^55^ ^]^ Briefly, mice were intraperitoneally injected with pentobarbital sodium (50 mg kg^−1^). For ABR recordings, three needle electrodes were inserted subcutaneously at the vertex of the head (reference), ipsilateral mastoid (recording), and contralateral rear leg of the mice (ground). ABR thresholds were recorded at 8, 12, 16, 20, 24, 28, and 32 kHz using a Tucker‐Davis Technologies (TDT) System and BioSigRZ software. The sound intensity varied from 90 to 20 dB, decreasing every 5 dB. The hearing threshold was defined as the lowest sound intensity required to generate a reproducible ABR waveform. DPOAE measurements at 2f1‐f2 were elicited from the test mice using BioSig‐RP software and a TDT system. Five frequency points (4, 8, 16, 28, and 32 kHz) were selected to measure 2f1‐f2(f2/f1 = 1.2) to predict the auditory thresholds. Hearing thresholds were defined as the average signal for each identified frequency tested and compared with the corresponding frequencies in the controls.

### Whole‐Mount Preparation of SGNs and Hair Cells

The cochleae were dissected from the temporal bone, fixed in 4% paraformaldehyde overnight at 4 °C, and then decalcified in 10% EDTA in PBS at 4 °C for 2–3 d. Decalcified cochleae were processed using 10% and 30% sucrose gradients and embedded in an OCT compound (Tissue‐Tek) for cryosectioning. The specimens were sliced into 10 µm sections for H&E staining and immunofluorescence. For hair cell preparation, the SGNs, Reissner's membrane, and the most basal cochlear segments were removed after fixation with 4% paraformaldehyde, followed by decalcification in 10% EDTA for 1–3 d at 4 °C. The hair cell preparations were used for immunofluorescence staining.

### Immunofluorescence

The cochlear specimens were washed three times with PBS. Permeabilization and blocking were conducted in a blocking buffer containing 20% goat serum with 0.3% TritonX‐100 and 3% BSA for 1 h at 37 °C, and then incubated with primary antibodies overnight at 4 °C. The specimens were washed three times with PBS and then incubated with secondary antibodies for 2 h at room temperature. Nuclei were counterstained with DAPI (#D9542; Sigma) for 10 min at room temperature. Finally, specimens were mounted with ProLong Gold antifade reagent (P36934; Thermo Fisher) and visualized using a confocal fluorescence microscope (Leica SP5, Microsystems, Germany). Primary antibodies used were, LRRC8A (sc‐517113; Santa Cruz Biotechnology, 1, 100) for hair cell and stria vascularis immunostaining (Figure 1D,F; Figures S1B, S2B,D, S4C,E, S6A, S10A,B, Supporting Information), LRRC8A (AAC‐001; Alomone labs, 1, 200) for SGNs (Figure 1E; Figures S2C, S4D, Supporting Information), Prestin (ab242128; Abcam, 1, 500), Myosin 7A (25‐679, Proteus, 1, 100), and Tuj1 (801 202; Biolegend, 1, 200). The secondary antibodies were Goat anti‐Rabbit (Alexa 488, 111‐545‐003, 1, 100), Goat anti‐Rabbit (Cyanine Cy3, 111‐165‐003, 1, 200), Goat anti‐Mouse (Alexa 488, 115‐545‐003, 1, 200), and Goat anti‐Mouse (Cyanine Cy3, 115‐165‐003, 1, 200), all from Jackson ImmunoResearch (USA).

Fluorescence intensity was quantified in immunostained images (Figure S1C–E, Supporting Information). For each age group, cochleae from three mice were analyzed, with 3–5 fields of view selected from apical, middle, and basal regions per mouse. Mean fluorescence intensity per cell was calculated for each field. Values were normalized to P1 baseline intensity to obtain relative fluorescence intensity.

### Cell Culture

HEK293A line (XB0421‐1808, Procell Tech, China) was maintained in DMEM with 10% FBS and a 1% penicillin‐streptomycin mixture (Gibco) at 37 °C with 5% CO_2_. The *Lrrc8a* knockout (KO) in HEK293A cells was generated using CRISPR/Cas9‐mediated genome editing (Beijing Biocytogen Co., Ltd, China). Briefly, exon 2 of the *Lrrc8a* gene was replaced with a puromycin resistance (puroR) cassette. Single‐guide RNA (sgRNA) was designed for vector cloning. The CRISPR/Cas9‐sgRNA complex was then delivered into HEK293A cells via electroporation. Single‐cell clones were selected using antibiotic screening and expanded. A successful knockout was verified by PCR genotyping and Western blot analysis. The plasmid containing the *SLC26A5* gene (NM_198 999.3, Youbio Biotech, China) was transiently transfected into HEK 293A cells at 500 ng mL^−1^ using Lipofectamine 2000 (Invitrogen). HEI‐OC1 mouse inner ear cell line (Biofeng Lab) was maintained in DMEM with 10% FBS at 33 °C with 10% CO_2_.

SGN cultures were prepared according to established protocols.^[^ ^55^ ^]^ Briefly, cochleae were dissected from temporal bones in a dissection solution containing minimum essential medium (MEM) supplemented with 0.2 g L^−1^ kynurenic acid, 10 mm MgCl_2_, 2% (v/v) fetal bovine serum (FBS), and 6 g L^−1^ glucose. The dissected cochleae were then enzymatically digested in a solution containing 1 mg mL^−1^ collagenase type I, 1 mg mL^−1^ DNase, and 2.5% (v/v) trypsin at 37 °C for 15 min. Following digestion, the cell suspension was centrifuged at 4491 g for 5 min in 0.45 m sucrose and resuspended in culture medium consisting of Neurobasal A supplemented with 2% (v/v) B27, and 0.5 mm L‐glutamine. The cell suspension was filtered through a 40 µm cell strainer and plated onto glass coverslips pre‐coated with 0.5 mg mL^−1^ poly‐D‐lysine and 1 mg mL^−1^ laminin. SGNs were maintained in a humidified incubator at 37 °C with 5% CO_2_ for 24 h before subsequent experiments.

### Quantitative Real‐Time PCR

Total RNA from mouse cochlea (4–5 mice were used for each sample) was extracted using an RNAeasy Micro Kit (Qiagen, Germany). According to the manufacturer's instructions, RNA was reverse‐transcribed at 37 °C for 30 min and followed by a step to 85 °C for 5 s to synthesize cDNA using the PrimeScript RT reagent Kit (Takara, China). Quantitative PCR was performed using the TB Green Kit (Takara) on a Bio‐Rad CFX Connect Real‐Time PCR system (Bio‐Rad Laboratories, USA). The protocol was performed at 95 °C for 30 s, followed by 40 cycles (95 °C for 5 s, 56 °C for 25 s, and 72 °C for 30 s), with a final elongation at 72 °C for 30 s. GAPDH was used as the reference gene. The relative mRNA expression levels were calculated using the standard formula 2^−∆∆Ct^. Table S2 (Supporting Information) lists the primer sequences.

### Single‐Cell Real‐Time PCR

SGNs and hair cells from WT mice were aspirated into a patch pipette using a patch‐clamp system under the microscope. The electrode tip containing cellular contents was rapidly transferred into a tube containing System 1 mixture (1 µL oligo‐dT primers, 1 µL dNTP mixture, and 2 µL RNase‐free water). The mixture was incubated at 65 °C for 5 min and immediately chilled on ice. Subsequently, System 2 mixture (1 µL PrimeScript II RTase, 0.5 µL RNase Inhibitor, 2 µL 5 × PrimeScript II buffer, and 1.5 µL RNase‐free water) was added to initiate reverse transcription. The reaction was incubated at 55 °C for 50 min, followed by enzyme inactivation at 85 °C for 5 min.

For cDNA amplification, a nested PCR approach was employed. The first‐round PCR was performed using 2 µL of cDNA template and the outer primer set. The second‐round PCR utilized the first PCR product as a template with an inner primer set. The PCR protocol consisted of an initial denaturation at 95 °C for 5 min, followed by 35 cycles of amplification (95 °C for 50 s, 59 °C for 50 s, 72 °C for 50 s), and a final extension at 72 °C for 10 min. PCR products were analyzed by 2% agarose gel electrophoresis. All reagents were obtained from commercial sources: PrimeScript RT reagent kit (6210A) from Takara‐Clontech (Invitrogen) and PCR reagents (M7122) from Promega (Madison, WI, USA). Table S3 (Supporting Information) lists the primer sequences.

### Electrophysiology

To record the VRAC current in OHCs, apical cochlear turns of P8–P12 mice were dissected in a solution containing (in mm) 140 NaCl, 5 KCl, 1 MgCl_2_, 0.1 CaCl_2_, 0.5 MgSO_4_, 10 HEPES, 3.5 L‐Glutamin, pH 7.3–7.4 adjusted with NaOH. Cells were continuously perfused with isotonic extracellular solutions containing (in mm), 140 NaCl, 5 KCl, 2 MgCl_2_, 2 CaCl_2_, 10 HEPES, 10 glucose, pH 7.3 adjusted with NaOH (300 mOsm kg^−1^). The hypotonic extracellular solution contained (mm), 95 NaCl, 5 KCl, 2 MgCl_2_, 2 CaCl_2_, 10 HEPES, 10 glucose, pH 7.3 adjusted with NaOH (220 mOsm kg^−1^). The pipette solution contained (mm), 130 CsCl, 2 MgCl_2_, 0.5 CaCl_2_, 10 HEPES, 5 EGTA, 5 ATP‐Mg, 0.5 GTP‐Na, pH 7.3 adjusted with CsOH. The VRAC currents were recorded at ‐84 mV stimulated by a hypotonic solution. A ramp protocol from ‐100 to 100 mV or a step voltage protocol ranging from ‐100 to 100 mV, with 20 mV increments, was also used to record currents. Recordings were performed using an Axon patch 700 B amplifier (Molecular Device, USA), a Digidata 1550 B digitizer, and the pClamp 10 software.

For the NLC recordings, the extracellular and pipette solutions were the same as those used in the VRAC current experiment. OHCs were held at –84 mV. Cell capacitance was measured using a continuous high‐resolution two‐sine wave stimulus protocol superimposed onto a voltage ramp from ‐150 to 120 mV.^[^ ^56^ ^]^ Capacitance data were fit to the first derivative of a two‐state Boltzmann function using Origin 7.5 software (OriginLab, USA) to calculate *Q_max_*, *V_1/2_*, *Charge Density* (CD), and *α* as follows,

(1) $$C(V)=C_{\mathrm{lin}}+\frac{Q_{\max}}{\alpha \exp\left[\left(V-V_{1/2}\right)/\alpha \right]{\left\{1+\exp\left[\left(V-V_{1/2}\right)/-\alpha \right]\right\}}^{2}}$$

(2) $$\mathrm{CD}\;=\frac{Q_{\max}/e^{-}}{C_{lin}/C_{conv}}$$

where *C* is the capacitance, and *V* is the voltage. *C_lin_* was calculated as the average capacitance at ‐150 mV and 120 mV. *CD* is the charge density or the number of electrons moved per square micrometer cell surface area, *e^−^* is the electron charge (1.602 × 10^−19^ C), and *C_conv_* is the specific membrane capacitance of 0.008 pF m^−2^. The NLC was calculated as the difference between the peak capacitance and linear capacitance. The NLC was recorded using a HEKA EPC10 patch‐clamp amplifier (Germany).

For outward current recording, OHCs were held at ‐84 mV with step voltage ranging from ‐144 to 36 mV, with 10 mV increments. The pipette solution contained (mm), 142 KCl, 3.5 MgCl_2_, 0.1 CaCl_2_, 5 HEPES, 1 EGTA, 2.5 ATP‐Mg, and 0.5 GTP‐Na, pH 7.3 adjusted with CsOH. The extracellular solutions contained (in mm), 140 NaCl, 5 KCl, 2 MgCl_2_, 2 CaCl_2_, 10 HEPES, 10 glucose, pH 7.3 adjusted with NaOH.

### Western Blot

Cells were harvested and homogenized in RIPA lysate containing a protease inhibitor cocktail. Protein concentration was determined using a BCA Protein Assay Kit (Solarbio, China). Equal amounts of protein were resolved by SDS‐PAGE and transferred to a PVDF membrane (Zomanbio, China). Membranes were blocked with 1 × TBST containing 5% dry milk for 2 h at room temperature and probed with anti‐LRRC8A (sc‐517113, Santa Cruz, 1, 200) and anti‐GAPDH (60004‐1, Proteintech, 1, 3000) overnight at 4 °C. Then, the membranes were incubated with secondary antibodies (IRDye 800CW goat anti‐rabbit, 1, 5000;IRDye 800CW goat anti‐mouse, 1, 5000;LI‐COR, Biosciences) for 90 min at room temperature. The immunoreactive bands were quantified using Image Lab 4.0 (Bio‐Rad, USA).

### Virus Injection

Hypothermia was used to induce and maintain sedation in neonatal mice at P6.^[^ ^57^ ^]^ A postauricular incision was made behind the auricle, followed by the exposure of the posterior semicircular canal (PSC) and lateral semicircular canal. The AAV‐8A rescue group mice (*Atoh1^Cre^;Lrrc8a^fl/fl^*) received PSC injections of AAV7m8‐CAG‐*Lrrc8a*‐P2a‐GFP (NM‐177725.4, Weizhen, China) in one ear. Control mice (*Atoh1^Cre^;Lrrc8a^fl/fl^*) were injected with AAV7m8‐CAG‐P2a‐GFP (Weizhen, China) under the same conditions. The ABR measurements were performed at P60.

### Measurement of Cell Volume

For cell volume measurement, OHCs were isolated from P10 to P14 mice using collagenase IV (100 U µL^−1^, Gibco) at 37 °C for 10 min. The isotonic extracellular solutions contain (in mm), 140 NaCl, 5 KCl, 2 MgCl_2_, 2 CaCl_2_, 10 HEPES, pH 7.3 adjusted with NaOH, (300 mOsm kg^−1^). The hypotonic extracellular solution contained (mm), 120 NaCl, 5 KCl, 1 MgCl_2_, 1 CaCl_2_, 10 HEPES, pH 7.3 adjusted with NaOH, (285 mOsm kg^−1^). The osmolarity was adjusted using mannitol. Single cells were immobilized in a Cell‐Tak‐coated chamber (3.5 mg cm^−2^; Corning). Cell imaging and volume measurements were performed using the Cellsens standard software. Length was defined as the distance between the apical and basal cell edges. The width represents the distance between the two lateral walls in the middle of the OHCs. The volume of the OHCs was estimated by considering the cell as a cylinder. Therefore, the volume of the OHCs is as follows^[^ ^58^ ^]^:

(3) $$\mathrm{Volume}=\;\frac{\pi \times \;\mathrm{width}\;\times \;\mathrm{width}\;\times \;\mathrm{length}}{4}$$

The cells were collected and resuspended to measure HEI‐OC1 cell volume. The cell volume was measured using a cell counting plate in a Live‐cell Dynamic Imaging and Analysis System (IncuCyte S3; Essen BioScience, USA). The average cell volume was *V* = 4/3*πR* ^3^, and the relative cell volume = *V_A_*/*V_C_*, with *V_C_* and *V_A_* representing the average cell volume in isotonic and hypotonic solutions, respectively.

### Statistical Analysis

All data were presented as the means ± standard error of mean (SEM). Statistical analyses were performed using the Origin software (version 8, USA) and GraphPad Prism software (version 8.0.2, USA). Student's *t*‐test was used to compare data between two groups, and one‐way analysis of variance (ANOVA) or two‐way ANOVA was used for multiple comparisons. *, **, and *** indicated statistically significant results compared with appropriate controls and indicated *p* < 0.05, *p* < 0.01, and *p* < 0.001, respectively.

## Conflict of Interest

The authors declare no conflict of interest.

## Author Contributions

S.W., Y.X., and Q.F. contributed equally to this work. P.L., H.S., and Z.X. designed the research, wrote, reviewed, and edited the manuscript; S.W., Y.X., Q.F., S.S., F.W., F.X., Z.Z., Y.Y., X.J., X.W., and C.C. performed the research and analyzed the data. H.Z. and N.G. reviewed and edited the manuscript.

## Supporting information



Supporting Information

## Data Availability

All data generated or analyzed during this study were included in the manuscript.
