# Supplementary material for: LRRC8A Regulates Outer Hair Cell Volume and Electromotility and is Required for Hearing
Source: Adv Sci (Weinh). 2025 Jul 11;12(41):e10477. doi: 10.1002/advs.202410477 (PMC12591118; doi:10.1002/advs.202410477)
Supplement: Supplementary file 1 — Supporting Information [file ADVS-12-e10477-s001.docx]

Supporting Information

**LRRC8A Regulates Outer Hair Cell Volume and Electromotility and is Required for Hearing**

Shengnan Wang^1*^, Yuehui Xi^2*^, Qiaojun Fang^3*^, Sai Shi^4^, Fei Wang^1^, Fuyu Xian^1^, Zhongyang Zhang^1^, Yuxin Yang^1^, Xishuo Jin^1^, Xiaomin Wang^1^, Chen Cao^1^, Hailin Zhang^1^, Nikita Gamper^1.5^, Zhigang Xu^2,6#^, Haitao Shen^7,8#^ and Ping Lv^1#^

^1^ Department of Pharmacology, The Key Laboratory of Neural and Vascular Biology, Ministry of Education, The Key Laboratory of New Drug Pharmacology and Toxicology, The Hebei Collaboration Innovation Center for Mechanism, Diagnosis and Treatment of Neurological and Psychiatric Disease, Hebei Medical University, Shijiazhuang, Hebei 050017, China.

^2^ Shandong Provincial Key Laboratory of Animal Cells and Developmental Biology and Key Laboratory for Experimental Teratology of the Ministry of Education, School of Life Sciences, Shandong University, Qingdao, Shandong 266237, China

^3^ Department of Otolaryngology-Head and Neck Surgery, The Second Affiliated Hospital of Anhui Medical University, Hefei 230601, China

^4^ Department of Medical and Pharmaceutical Informatics, Hebei Medical University, Shijiazhuang, Hebei 050017, China

^5^ Faculty of Biological Sciences, School of Biomedical Sciences, University of Leeds, Leeds, United Kingdom

^6^ Department of Otolaryngology Head and Neck Surgery, the First Hospital of Lanzhou University, Lanzhou, Gansu 730000, China

^7^ Lab of Pathology, Hebei Medical University, Shijiazhuang, Hebei 050017, China

^8^ Hebei Collaborative Innovation Center of Tumor Microecological Metabolism Regulation, Affiliated Hospital of Hebei University, Baoding, Hebei, China


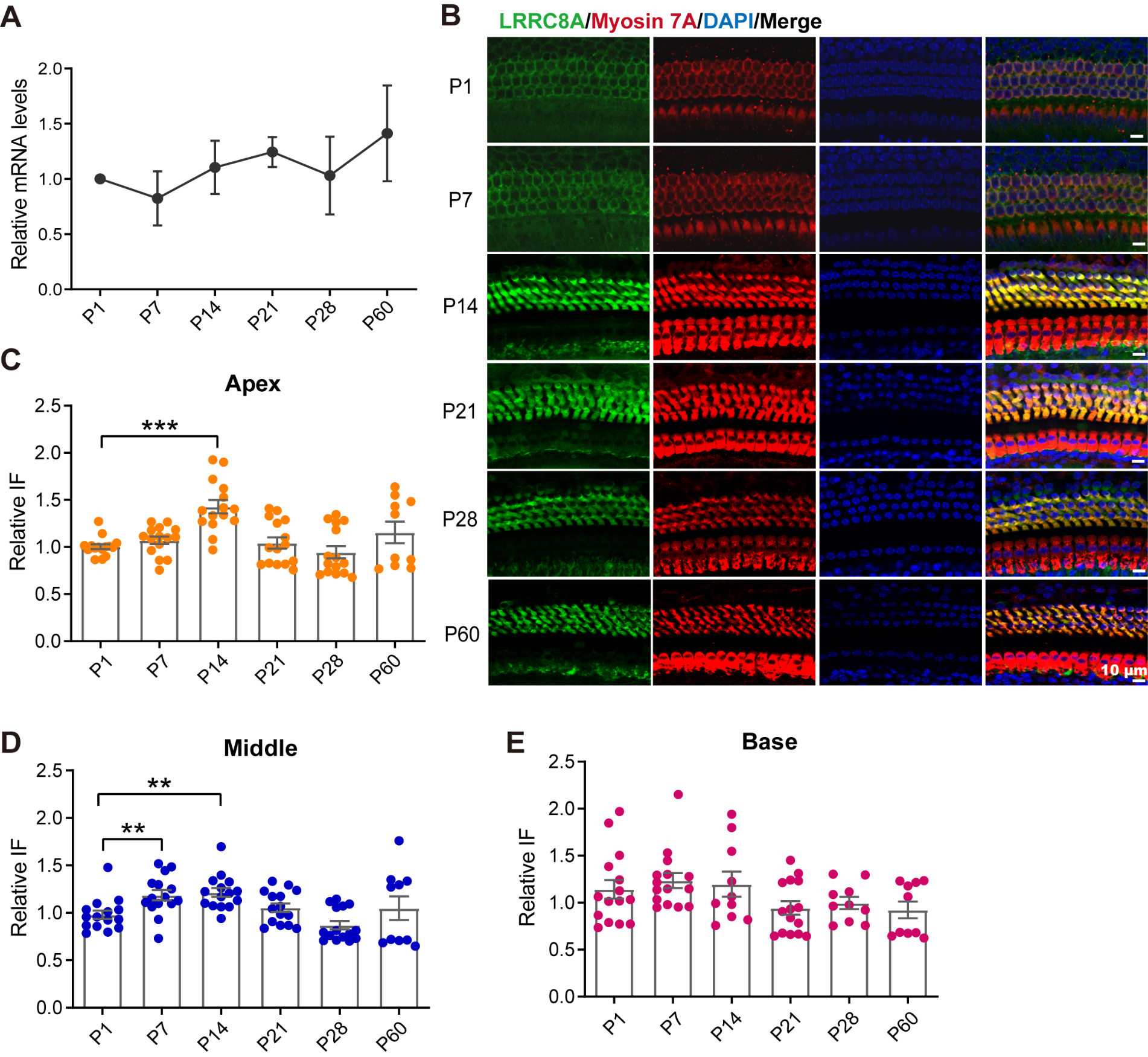


**Fig. S1 Expression of LRRC8A in OHCs during cochlea development**

(**A**) Relative expression of LRRC8A in the cochlea at different developmental stages was determined by real-time PCR (*n*=3/group). (**B**) Confocal images showing the LRRC8A expression (green) in hair cells of WT mice at different developmental stages. Hair cells were indicated by Myosin 7A (red) immunoreactivity. Scale bar: 10 μm. (**C**–**E**) Quantification of LRRC8A fluorescence intensity in OHCs at apical (C), middle (D) and basal turns (E) of cochlea at different developmental stages. Data are means ± SEM, ***p* < 0.01, ****p* < 0.001 by one-way ANOVA.


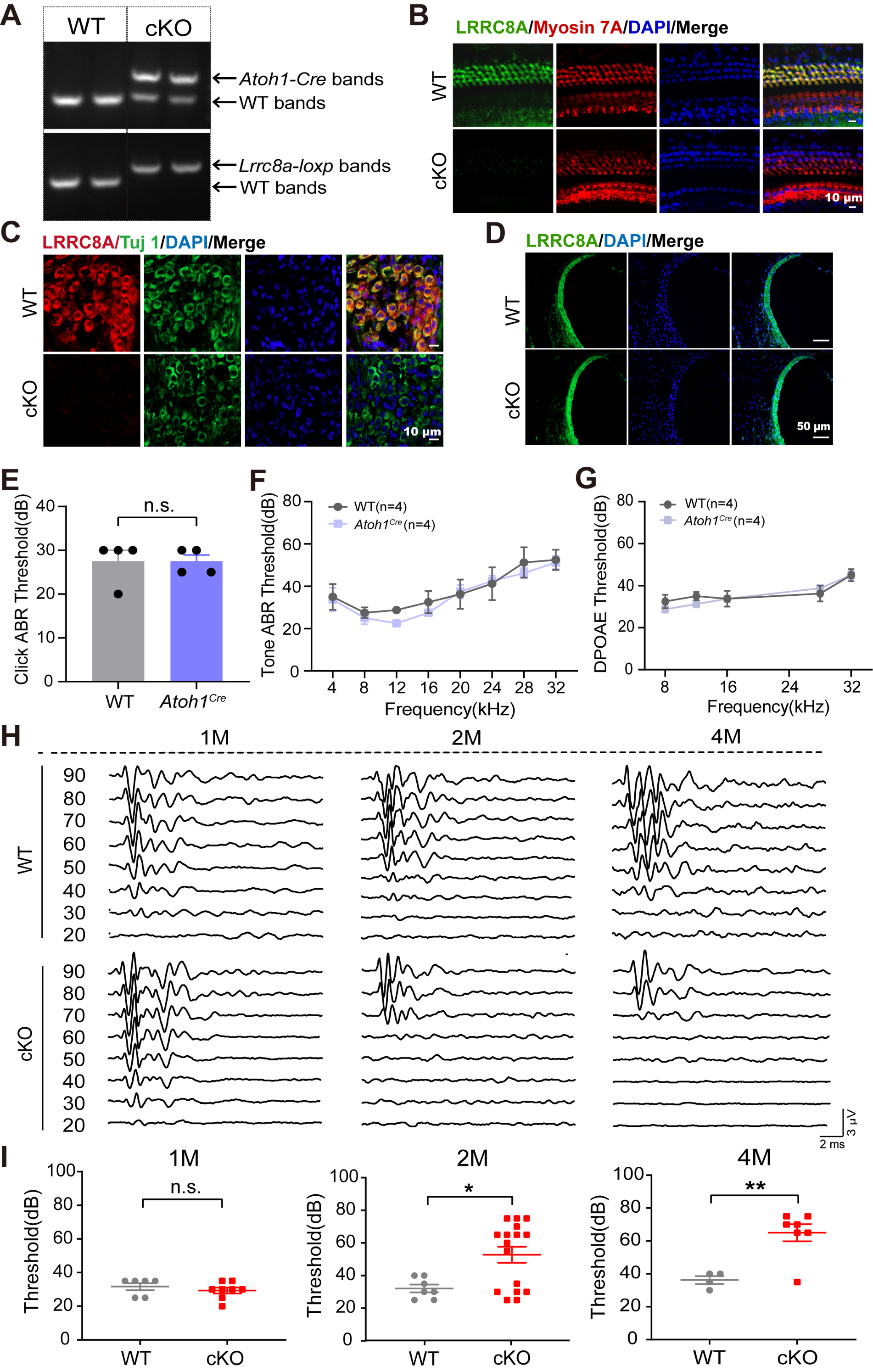


**Fig. S2** **LRRC8A expression and ABR thresholds in WT and *Lrrc8a* cKO mice**

(**A**) PCR genotyping of WT and *Lrrc8a* cKO mice using genomic DNA prepared from tail biopsies. *Lrrc8a* cKO mice were identified by the *Lrrc8a* target band and *Atoh1* Cre target band. (**B**–**D**) Immunostaining of LRRC8A in hair cells (B), SGNs (C), and stria vascularis (D) from 2-month-old WT and cKO mice. Antibodies against Myosin 7A and Tuj1 were used to label hair cells and SGNs, respectively. (**E, F**) ABR thresholds to click (E) and pure tone stimuli (F) in WT and *Atoh1^Cre^* mice at 2 months of age. (**G**) DPOAE thresholds to pure tone stimuli in 2-month-old WT and *Atoh1^Cre^* mice. (**H**) Representative ABR waveforms in response to click stimuli in 1-, 2-, and 4-month-old WT and cKO mice. (**I**) Thresholds statistics of WT and cKO mice at 1, 2, and 4 months of age in response to click stimuli. Data are means ± SEM, **p* < 0.05, ***p* < 0.01 by Student’s *t*-test (E, I) and two-way ANOVA (F, G).


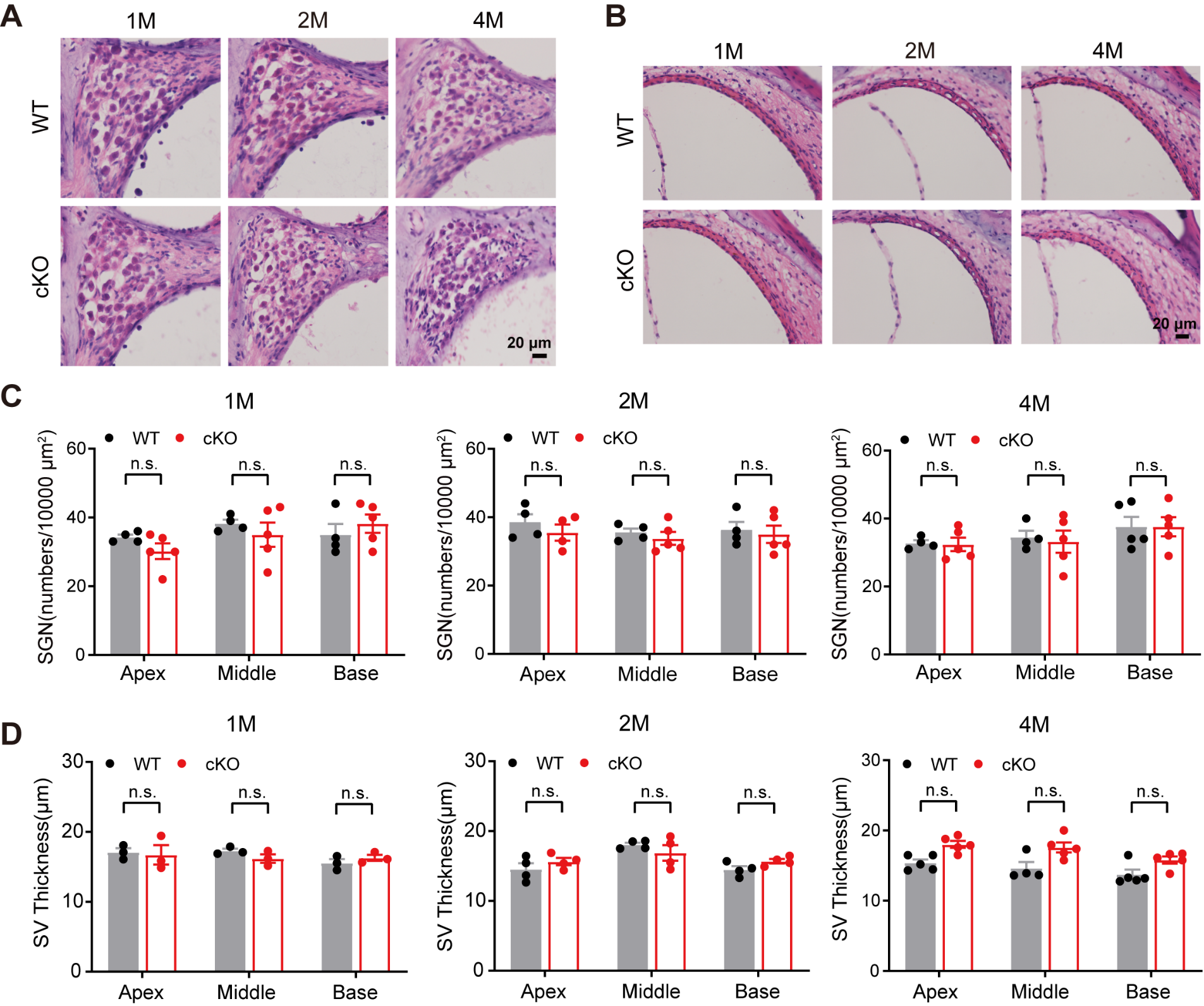


**Fig. S3 Morphometry changes in SGNs and stria vascularis (SV) in cKO mice**

**(A)** Morphometry changes in SGNs in the basal cochlea of WT and cKO mice. *n*= 4/4/4 for WT 1M/2M/4M, *n*=5/5/5 for cKO 1M/2M/4M. **(B)** Morphometry changes in SV in the basal cochlea of WT and cKO mice. *n* = 3/4/5 for WT 1M/2M/4M, *n* = 3/4/5 for cKO 1M/2M/4M. **(C)** SGN numbers were quantified in all three regions of the cochlea according to results similar to (A). **(D)** SV thickness was quantified in all three regions of the cochlea according to results similar to (B). Data are means ± SEM. Two-way ANOVA was used for analysis.


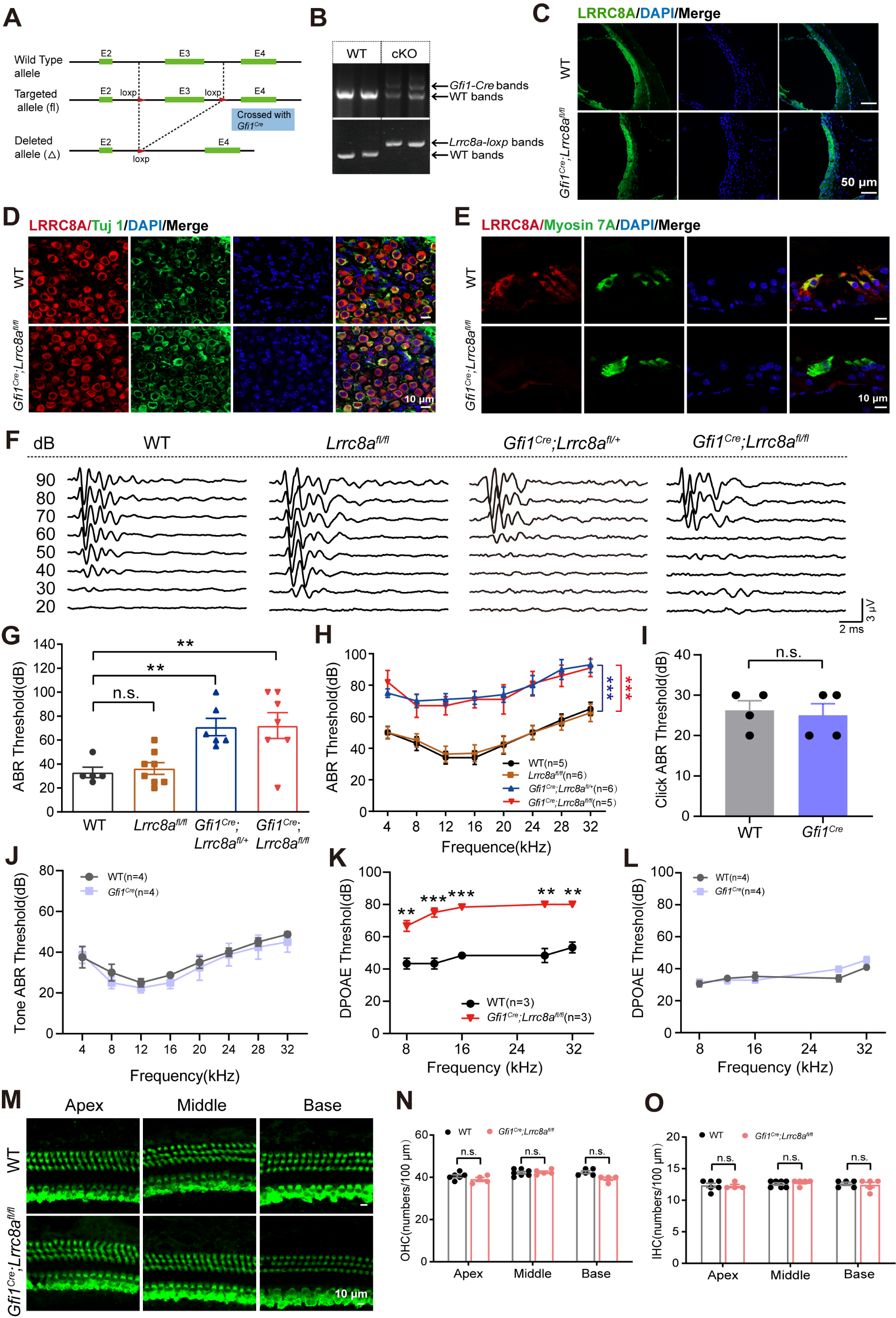


**Fig. S4 Hair cell-specific LRRC8A knockout mice exhibit hearing loss and OHC dysfunction**

(**A**) Schematic diagram of the strategy for *Gfi1^Cre^;Lrrc8a^fl/fl^* mouse construction. Two loxP sites were inserted into the *Lrrc8a* alleles, flanking the coding sequence of exon 3. Cre recombinase expression is driven by the specific *Gfi1* promoter in the hair cells to delete the exon 3 region between the homodromous loxP sites, resulting in LRRC8A function loss in hair cells. (**B**) PCR genotyping of WT and *Gfi1^Cre^;Lrrc8a^fl/fl^* mice using genomic DNA prepared from tail biopsies. *Gfi1^Cre^;Lrrc8a^fl/fl^* mice were identified by the *Lrrc8a* target band and *Gfi1^Cre^* target band. (**C**–**E**) Immunostaining of LRRC8A in stria vascularis (C), SGNs (D), and hair cells (E) from 2-month-old WT and *Gfi1^Cre^*;*Lrrc8a^fl/fl^* mice. Antibodies against Myosin 7A and Tuj1 were used to label hair cells and SGNs, respectively. (**F**) Representative ABR waveforms in response to click stimuli in 2-month-old WT, *Lrrc8a^fl/fl^,* *Gfi1^Cre^;Lrrc8a^fl^*^/+^, and *Gfi1^Cre^;Lrrc8a^fl/fl^* mice. (**G, H**) ABR thresholds to click (G) and pure tone stimuli (H) were measured in WT, *Lrrc8a^fl/fl^*, *Gfi1^Cre^;Lrrc8a^fl^*^/+^, and *Gfi1^Cre^;Lrrc8a^fl/fl^* mice at 2 months of age. (**I, J**) ABR thresholds to click (I) and pure tone stimuli (J) were measured in WT and *Gfi1^Cre^* mice. (**K**, **L**) DPOAE threshold measurement of 2-month-old WT and *Gfi1^Cre^;Lrrc8a^fl/fl^* mice (K) and WT and *Gfi1^Cre^* mice (L). (**M**) Myosin 7A-positive hair cells from 2-month-old WT and *Gfi1^Cre^;Lrrc8a^fl/fl^* mice. Scale bar: 10 μm. (**N, O**) Quantification of OHCs (N) and IHCs (O) at the cochlea’s apical, middle, and basal regions in WT and *Gfi1^Cre^; Lrrc8a^fl/fl^* mice. *n*=6/7/5 for WT apical/middle/basal cochlea, *n*=4/5/5 for *Gfi1^Cre^;Lrrc8a^fl/fl^* apical/middle/basal cochlea. Data are means ± SEM, **p*<0.05, ***p*<0.01, ****p*<0.001 by Student’s *t*-test (I), one-way ANOVA (G), and two-way ANOVA (H, J–L, N, O).


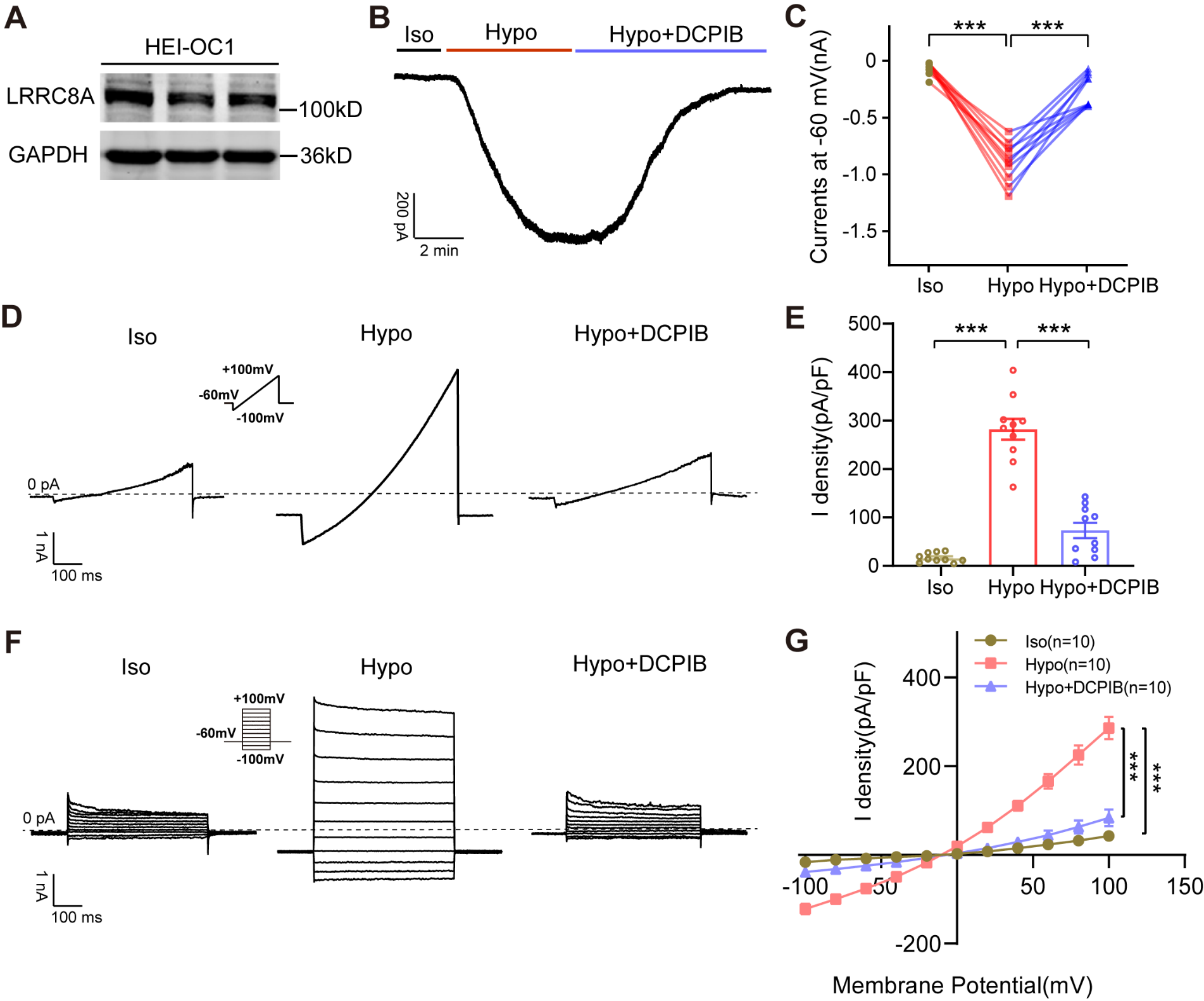


**Fig. S5 VRAC mediate swelling-activated currents in HEI-OC1 cells**

(**A**) Expression of LRRC8A in HEI-OC1 cells was examined by performing Western blotting using anti-LRRC8A antibody. GAPDH was included as internal control. (**B**) Representative current traces recorded from HEI-OC1 cells induced by Iso (300 mOsm kg^-1^) and Hypo (220 mOsm kg^-1^) solutions with and without the presence of 10 μM DCPIB at -60 mV. (**C**) Quantification of current at -60 mV under isotonic (Iso), hypotonic (Hypo), and DCPIB-treated hypotonic conditions (Hypo+DCPIB). (**D)** Representative whole-cell currents recorded by ramp protocol under Iso, Hypo, and Hypo+DCPIB conditions. (**E**) Quantification of current densities at +100 mV in ramp protocol under Iso, Hypo and Hypo+DCPIB conditions. (**F**) Representative whole-cell currents recorded by voltage step under Iso, Hypo, and Hypo+DCPIB conditions. (**G**) The current density–voltage relationship under Iso, Hypo, and Hypo+DCPIB conditions. Data are means ± SEM, ****p* < 0.001 by one-way ANOVA (C, E) and two-way ANOVA (G).


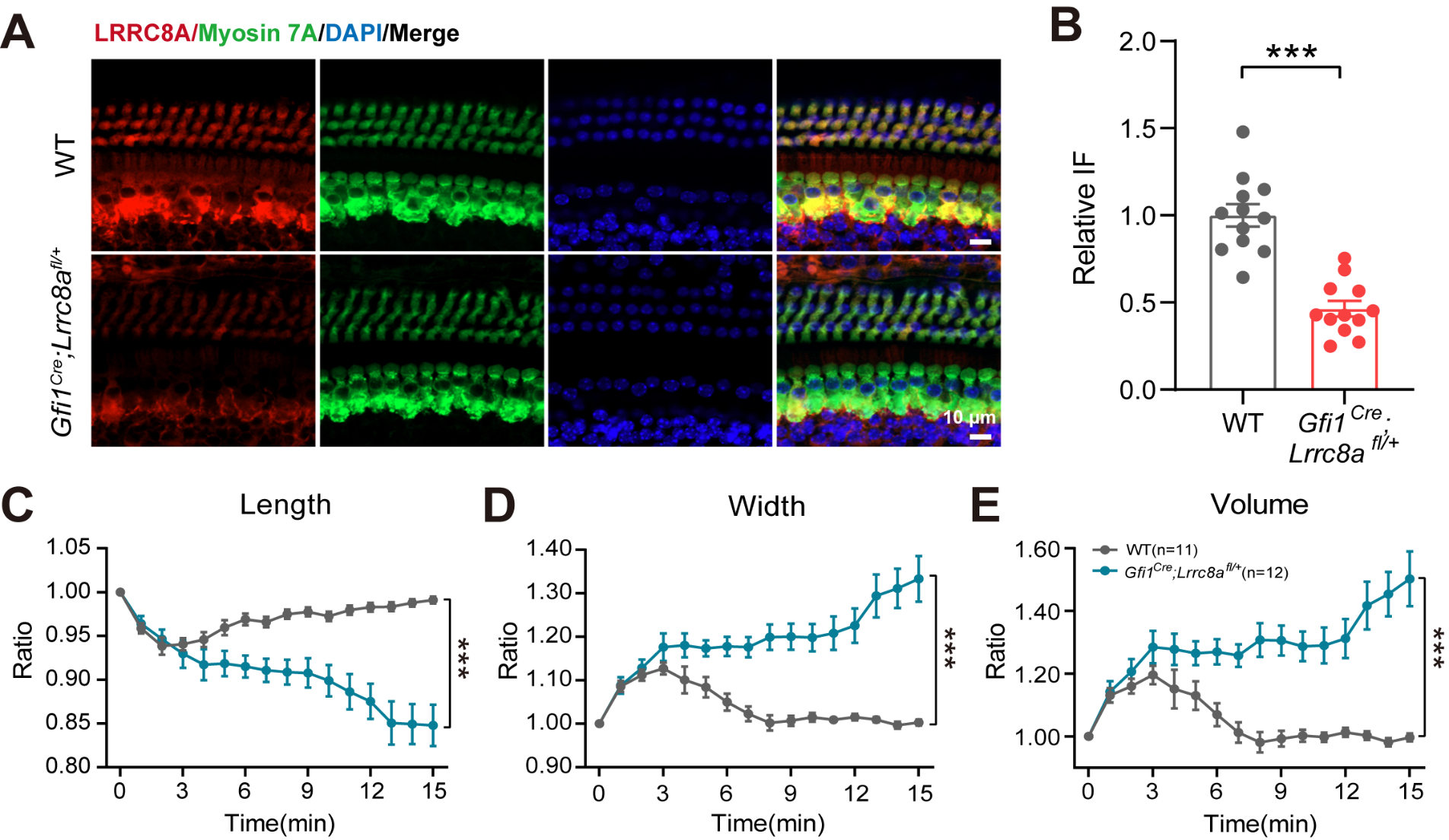


**Fig. S6 OHC volume regulation in *Gfi1^Cre^;Lrrc8a^fl^*^/+^ mice**

(**A**) Immunostaining of LRRC8A in hair cells from 2-month-old WT and *Gfi1^Cre^*;*Lrrc8a^fl/+^* mice. Antibodies against Myosin 7A were used to label hair cells. (**B**) Quantification of fluorescence intensity of LRRC8A in hair cells from WT and *Gfi1^Cre^*;*Lrrc8a^fl/+^* mice. (**C-E**) Quantification of changes in length (C), width (D), and volume (E) of isolated OHCs from WT and *Gfi1^Cre^*;*Lrrc8a^fl/+^* mice treated with hypotonic solution (285 mOsm kg^-1^). Data are means ± SEM, ****p* < 0.001 by Student’s *t*-test (B) and two-way ANOVA (C-E).


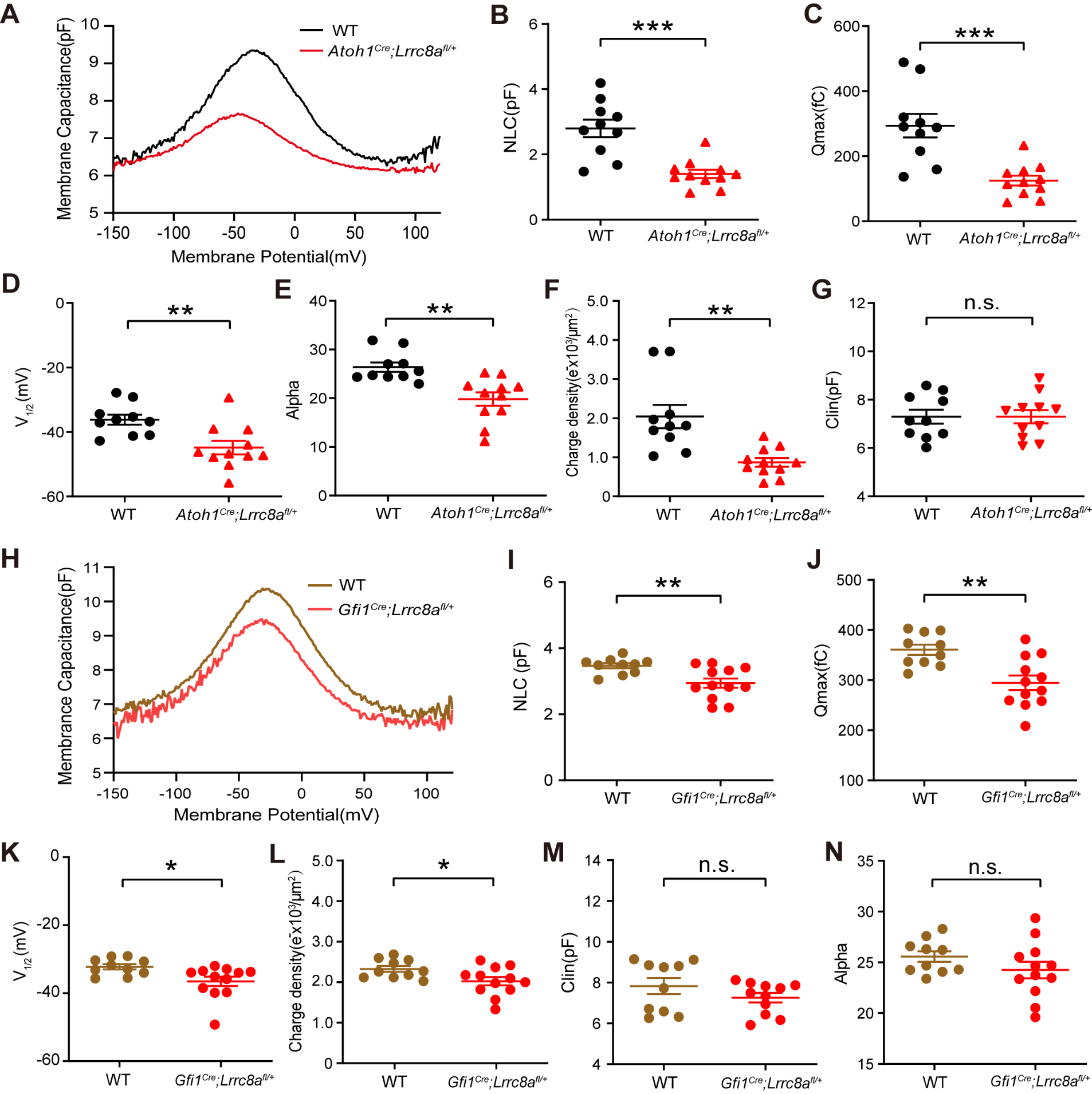


**Fig. S7** **OHC electromotility in** ***Atoh1^Cre^;Lrrc8a^fl^*^/+^ and *Gfi1^Cre^;Lrrc8a^fl^*^/+^ mice**

(**A**) Voltage-dependent capacitance curves in OHCs from WT and *Atoh1^Cre^;Lrrc8a^fl^*^/+^ mice at P8–P12. (**B**) Quantification of NLC in OHCs from WT and *Atoh1^Cre^;Lrrc8a^fl^*^/+^ mice. (**C**–**G)** Electromotility parameters in OHCs from WT and *Atoh1^Cre^;Lrrc8a^fl^*^/+^ mice: Q_max_ (C), V_1/2_ (D), α (E), charge density (F), and C_lin_ (G). (**H**) Voltage-dependent capacitance curves in WT and *Gfi1^Cre^;Lrrc8a^fl^*^/+^ mice at P8-P12. (**I**) Quantification of NLC in OHCs from WT and *Gfi1^Cre^;Lrrc8a^fl^*^/+^ mice. (**J**–**N)** Electromotility parameters in OHCs from WT and *Gfi1^Cre^;Lrrc8a^fl^*^/+^ mice: Q_max_ (J), V_1/2_ (K), charge density (L), C_lin_ (M), and α (N). Data are means ± SEM, **p* < 0.05, ***p* < 0.01, ****p* < 0.001 by Student’s *t*-test.


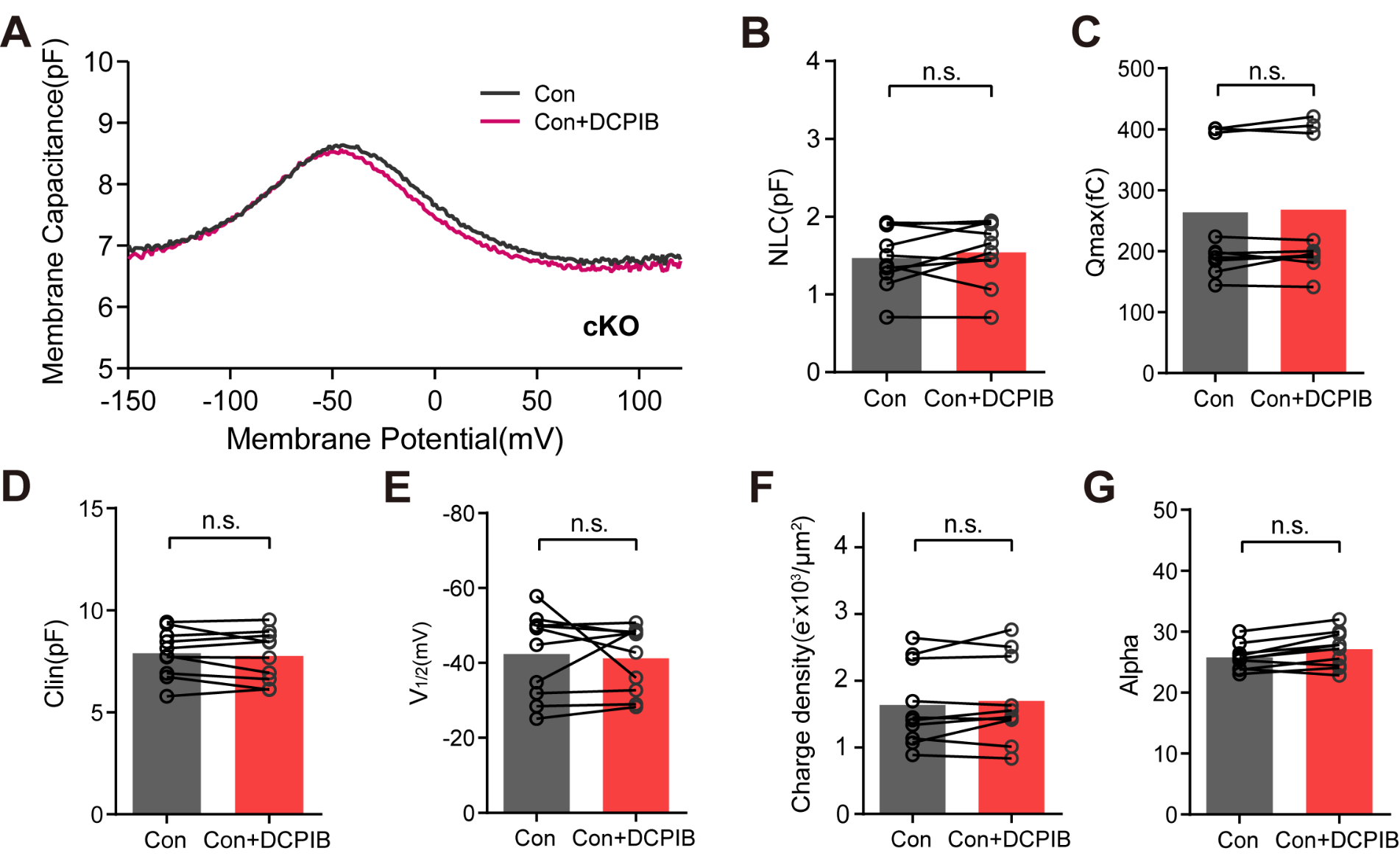


**Fig. S8** **Effects of DCPIB on OHC electromotility in cKO mice**

(**A**) Voltage-dependent capacitance curves in cKO OHCs from control and DCPIB-treated (10 μM) groups. (**B**) Quantification of NLC in control versus DCPIB-treated cKO OHCs. **(C**–**G)** Electromotility parameters in control and DCPIB-treated cKO OHCs: Q_max_ (C), C_lin_ (D), V_1/2_ (E), charge density (F), and α (G). Data are means ± SEM. Student’s *t*-test was used for analysis.


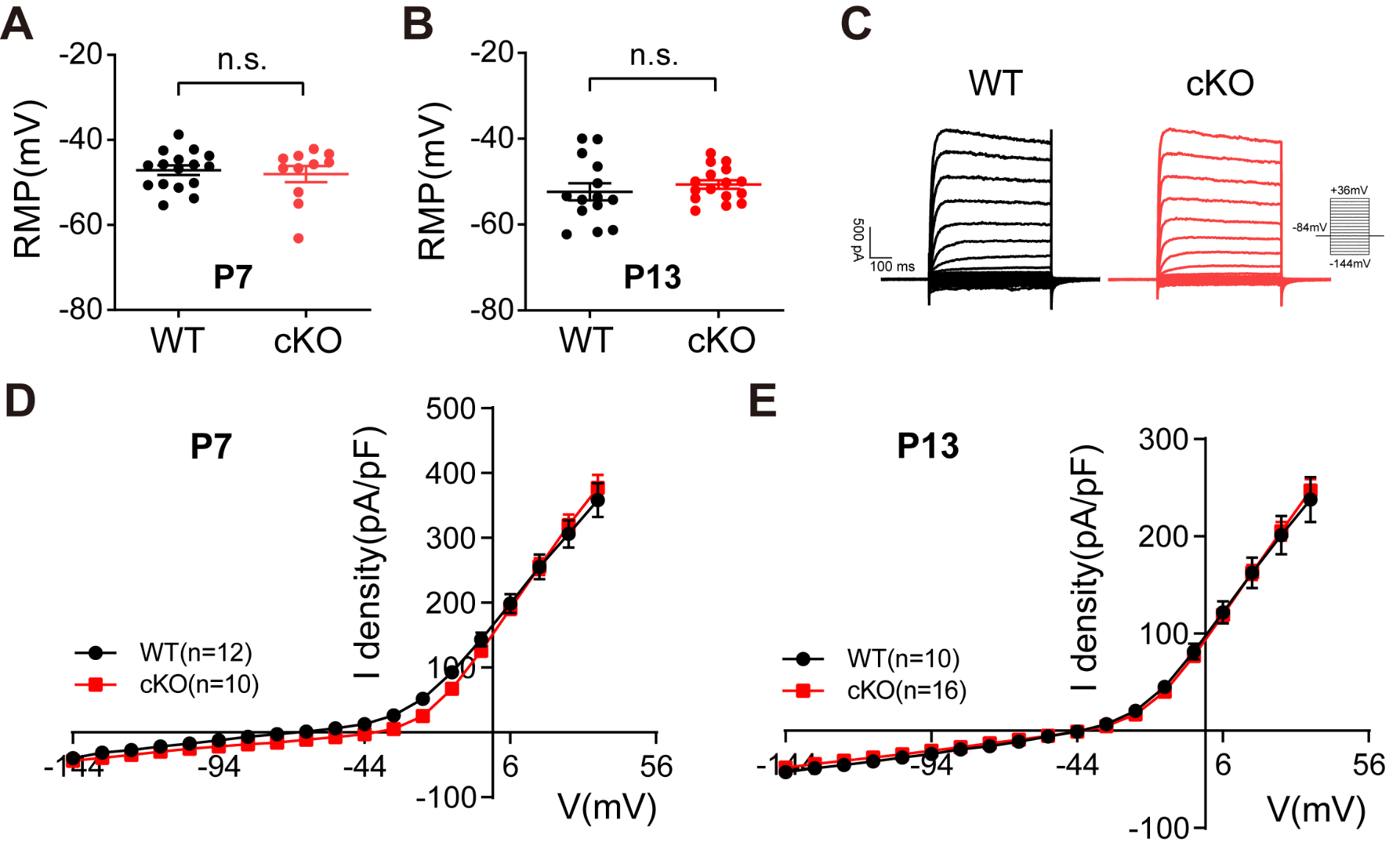


**Fig. S9 Knockout of LRRC8A did not affect resting membrane potential and outward current of OHCs**

(**A, B**) Resting membrane potential (RMP) of OHCs from WT and cKO mice at P7 (A) and P13 (B). (**C**) Current responses from OHCs of WT and LRRC8A cKO mice at P7 elicited by applying depolarizing voltage steps from -144 mV to +36 mV with 10 mV increments. **(D, E**) Current density–voltage relationships of outward currents recorded from OHCs of WT and cKO mice at P7 (D) and P13 (E). Data are means ± SEM. Student’s *t*-test (A, B) and two-way ANOVA (D, E) were used for analysis.


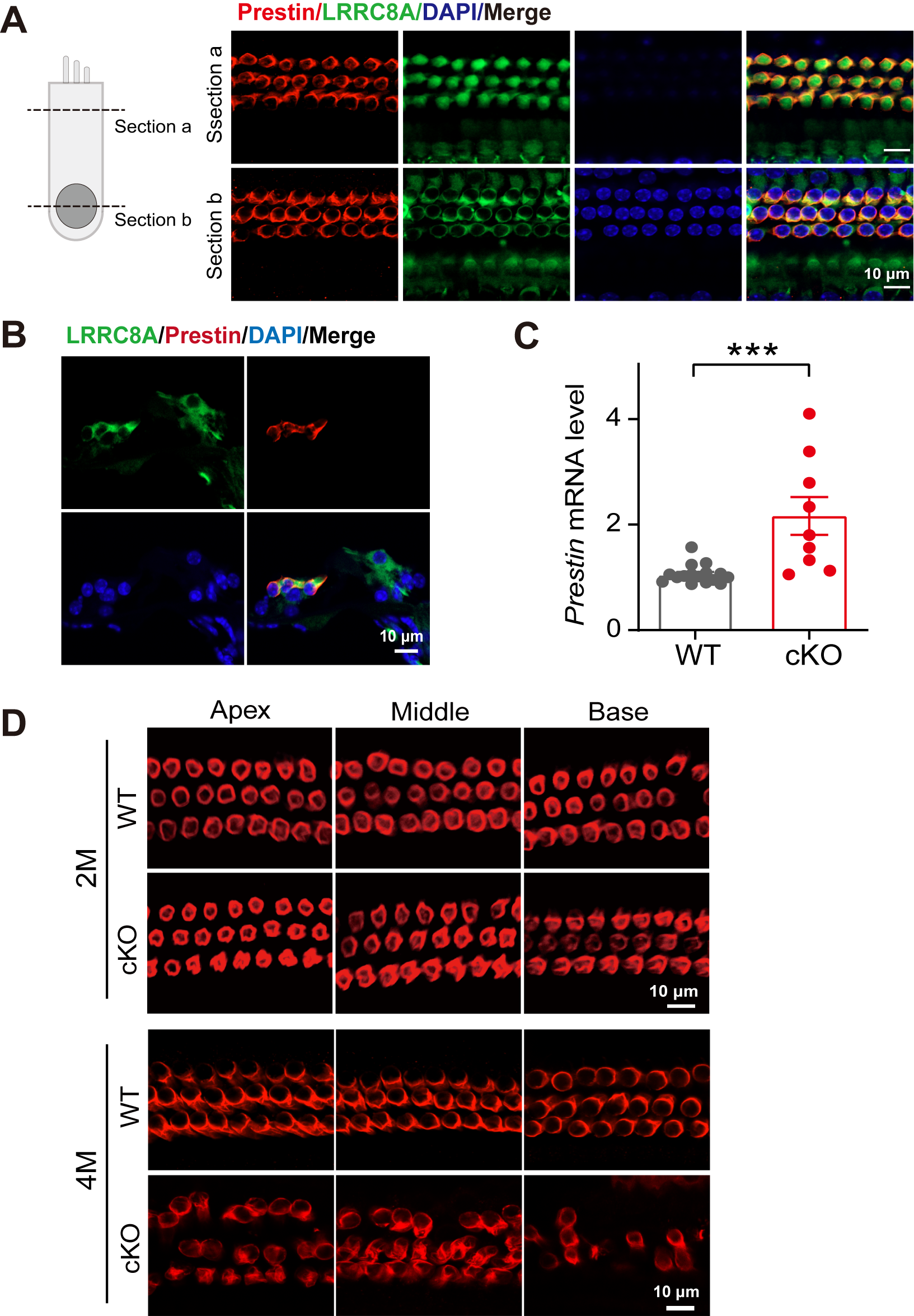


**Fig. S10 Loss of LRRC8A affects Prestin expression in OHCs**

(**A**) Immunofluorescence analysis for LRRC8A (green) and Prestin (red) in nuclear or cytoplasmic levels of OHCs from 2-month-old WT mice. Schematic on the left depicts focal planes for images shown on the right. (**B**) Immunostaining revealed that LRRC8A (green) is partially overlapped with Prestin (red) in OHCs from 2-month-old WT mice. The nuclei were stained with DAPI (blue). (**C**) The Prestin mRNA level was increased in cKO mice, as detected by RT-PCR. **(D)** Prestin expression in OHCs from 2-, and 4-month-old WT and cKO mice. Data are means ± SEM, ****p*<0.001 by Student’s *t*-test.

**Table S1. The primer sequences for genotyping**

| Primer | Sequence (5′-3′) |
| --- | --- |
| LRRC8A forward | GCTTAGGCTCCTTGGCAGAATGTCA |
| LRRC8A reverse | AGGTGTAGACAGGGCTATGGAGAGAC |
| Atoh1 forward | CCGGCAGAGTTTACAGAAGC |
| Atoh1 reverse | ATGTTTAGCTG GCCCAAATG |
| Atoh1 control forward | CTAGGCCACAGAATTGAAAGATCT |
| Atoh1 control reverse | GTAGGTGGAAATTCTAGCATCATCC |
| Gfi1 WT forward | CCGAGGGGCGTTAGGAT |
| Gfi1 Cre forward | GCCCAAATGTTGCTGGATAGT |
| Gfi1 common reverse | GGGATAACGGACCAGTTG |

**Table S2. The primer sequences for quantitative real-time PCR**

| **Primer** | **Sequence (5′-3′)** |
| --- | --- |
| LRRC8A forward | TCAGCAAGATGAACGGGTCC |
| LRRC8A reverse | CCCTGCTCAATCCGTACTT |
| LRRC8B forward | TTCCGGTTGACCAGCATTCA |
| LRRC8B reverse | TCCCTTGTAGAGAGGAGAGGTA |
| LRRC8C forward | CCCTCGCCCCCAGAGATTA |
| LRRC8C reverse | ATCCGTGAACACATCCCACC |
| LRRC8D forward | GTCCCCCTTTATGTGGGGTA |
| LRRC8D reverse | GTCAGCAGTGCACTTCCTCT |
| LRRC8E forward | CCTGAGCAGTGAGCAGGATG |
| LRRC8E reverse | ATGAGCATAGCCACGGTGAG |
| TMEM16A forward | TTGATAACCCTGCCACCGTC |
| TMEM16A reverse | CCTTGACAGCTTCCTCCTCC |
| TMEM16B forward | ACAATCCAGCCACCGTCTTC |
| TMEM16B reverse | TGTTCCTGGGAACGTTCTTCTT |
| TMEM16F forward | CAAATGGAGGAGGAGGAGGAC |
| TMEM16F reverse | GGTGCGTGTACTTTTACAAATAC |
| TMEM63B forward | TGCGCCTAGGGGAGGAT |
| TMEM63B reverse | TTCGGGTTGCTGCTGTTGA |
| CIC1 forward | GGTGTCTATGAGACCGTGCC |
| CIC1 reverse | TCCATCAGGGGCTGCAAAG |
| CIC2 forward | CAGCACATGCAAAAGCTAAGAAAA |
| CIC2 reverse | GCGGATAGATGTCTCGGAGCTA |
| CIC-K1 forward | GGCAGGACCTCTACAGGGTG |
| CIC-K1 reverse | CCACTCTAGGCCCCCTCTGAT |
| CIC-K2 forward | CAGGGCACACTGACAGAGAA |
| CIC-K2 reverse | CCCTGGATGTTTCTGCGGAT |
| CFTR forward | TCTGCCGCGCAGCAA |
| CFTR reverse | GGTGTGAACGTCATCAGATCCA |
| GAPDH forward | TTGATGGCAACAATCTCCAC |
| GAPDH reverse | CGTCCCGTAGACAAAATGGT |

**Table S3. The primer sequences for single cell RT-PCR**

| **Primer** | **Sequence 5′-3′** | |
| --- | --- | --- |
| Outer primers  LRRC8A forward | GATAGCAGAGTCATCTCAGGGTTA | |
| LRRC8A reverse | TTGATGATGGTCTGCCGCAT | |
| LRRC8B forward | TGCTGTGACCAACAATAACATTGAG | |
| LRRC8B reverse | ATGTCCTAGAACCCCTGGCT | |
| LRRC8C forward | CCTCGCCCCCAGAGATTA | |
| LRRC8C reverse | CCGCAGCAGATTTGTCAACC | |
| LRRC8D forward | GCCCGCGAAGGAAGTG | |
| LRRC8D reverse | TGCTGGGAACTTCGATCACC | |
| LRRC8E forward | GTCTGTAGTGAGGCGGGGC | |
| LRRC8E reverse | TGATCTTGTCCTGTGTCACCTGG | |
| GAPDH forward | TTAAGAGGGATGCTGCCCTTA | |
| GAPDH reverse  Inner primers | CATTGCTGACAATCTTGAGTGAGTT | |
| LRRC8A forward | | AGCCTTGGTGGGATGTGTTC |
| LRRC8A reverse | | TGAGTCGAAGCACTTGAGCA |
| LRRC8B forward | | GATTGAGGGGCGTGTCGTTA |
| LRRC8B reverse | | TCAAAGCCAGTGGTGAGCAT |
| LRRC8C forward | | CCTCGCCCCCAGAGATTA |
| LRRC8C reverse | | CTTGGCATACCAGTGGAGGG |
| LRRC8D forward | | GTCGATGGGGGACACTCTGT |
| LRRC8D reverse | | CAGCAGTGCACTTCCTCTCCTC |
| LRRC8E forward | | GCCCTGACTCACCCTCTCT |
| LRRC8E reverse | | GGGATCATCCTGCTCACTGC |
| GAPDH forward | | CCAGCCTCGTCCCGTAGACA |
| GAPDH reverse | | CTCGTGGTTCACACCCATCA |
